# Disrupting EDEM3‐induced M2‐like macrophage trafficking by glucose restriction overcomes resistance to PD‐1/PD‐L1 blockade

**DOI:** 10.1002/ctm2.70161

**Published:** 2025-01-03

**Authors:** Shaoyong Peng, Minshan Wu, Qian Yan, Gaopo Xu, Yumo Xie, Guannan Tang, Jinxin Lin, Zixu Yuan, Xiaoxia Liang, Ze Yuan, Jingrong Weng, Liangliang Bai, Xiaolin Wang, Huichuan Yu, Meijin Huang, Yanxin Luo, Xiaoxia Liu

**Affiliations:** ^1^ Department of General Surgery (Colorectal Surgery) The Sixth Affiliated Hospital Sun Yat‐sen University Guangzhou Guangdong China; ^2^ Guangdong Institute of Gastroenterology Guangzhou Guangdong China; ^3^ Guangdong Provincial Key Laboratory of Colorectal and Pelvic Floor Diseases The Sixth Affiliated Hospital Sun Yat‐sen University Guangzhou Guangdong China; ^4^ Biomedical Innovation Center The Sixth Affiliated Hospital Sun Yat‐sen University Guangzhou Guangdong China; ^5^ Key Laboratory of Human Microbiome and Chronic Diseases (Sun Yat‐sen University) Ministry of Education Guangzhou China; ^6^ School of Life Sciences Innovation Center of the Sixth Affiliated Hospital Sun Yat‐sen University Guangzhou China; ^7^ Department of Medical Oncology The Sixth Affiliated Hospital Sun Yat‐sen University Guangzhou Guangdong China; ^8^ School of Pharmaceutical Sciences Guangzhou University of Chinese Medicine Guangzhou Guangdong China

**Keywords:** 2‐DG, antitumour immunity, cancer‐associated fibroblasts, fasting‐mimicking diet, glycosyltransferase EDEM3, M2‐like macrophage, PD‐L1 glycosylation

## Abstract

**Background:**

Immunotherapy is beneficial for some colorectal cancer (CRC) patients, but immunosuppressive networks limit its effectiveness. Cancer‐associatedfibroblasts (CAFs) are significant in immune escape and resistance toimmunotherapy, emphasizing the urgent need for new treatment strategies.

**Methods:**

Flow cytometric, Western blotting, proteomics analysis, analysis of public database data, genetically modified cell line models, T cell coculture, crystal violetstaining, ELISA, metabonomic and clinical tumour samples were conducted to assess the role of EDEM3 in immune escape and itsmolecular mechanisms. We evaluated theeffects of FMD plus 2‐DG on antitumour immunity using multipleximmunofluorescence, flow cytometry, cytokine profiling, TUNEL assays, xenografttumours, and in vivo studies.

**Results:**

We show thatCAFs upregulate PD‐L1 glycosylation and contribute to immune evasion byglycosyltransferase EDEM3. Additionally, EDEM3 plays a role in tumour immunityduring tumour progression. However, the EDEM3‐mediated upregulation of PD‐L1 expression underpins PD‐1/PD‐L1 blockade resistance in vivo. This finding contradictsthe previous trend that positive PD‐L1 expression indicates a strong responseto PD‐1/PD‐L1 blockade. Mechanistically, high‐EDEM3 expression facilitates M2‐like This finding contradictsthe previous trend that positive PD‐L1 expression indicates a strong responseto PD‐1/PD‐L1 blockade.Mechanistically, polarizationand chemotactic migration of macrophages, which are enriched in theperipheral region of tumours compared to thecore region, precluding access of CD8+ T cells to tumourfoci. Furthermore, we EDEM3 predominantly activates the recruited M2‐like macrophagesvia a glucose metabolism‐dependent mechanism. Manipulationof glucose utilization by a fasting‐mimicking diet(FMD) plus 2‐DG treatmentsynergistically with PD‐1 antibody elicits potent antitumour activity byeffectively decreasing tumour glycosylated PD‐L1 expression, augmenting the CD8+effector T cell infiltration and activation while concurrently reducing the infiltration.TheCAFs‐EDEM3‐M2‐like macrophage axis plays a critical role in promotingimmunotherapy resistance. infiltration.TheCAFs‐EDEM3‐M2‐like macrophage axis plays a critical role in promotingimmunotherapy resistance.

**Conclusions:**

Our study suggests that blocking EDEM3‐induced M2‐like macro phage trafficking by FMD plus 2‐DG is a promising and effective strategy to overcomeresistance to checkpoint blockade therapy offeringhope for improved treatment outcomes.

**Key points:**

- Cancer‐associated fibroblasts (CAFs) can enhance PD‐L1 glycosylation through the glycosyltransferase EDEM3, contributing to immune evasion during tumour progression.
- EDEM3 predominantly activates the recruit M2‐like macrophages via a glucose metabolism‐dependent mechanism.
- Blocking glucose utilization antagonizes recruiting and polarizing M2‐like macrophages synergistically with PD‐1 antibody to improve anticancer immunity.

## Graphical abstract

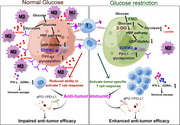

## 1 INTRODUCTION

The prevalence and disease burden of colorectal cancer (CRC) is rising globally.^1^ CRC is the second leading cause of death worldwide, following lung cancer.^2^ A systematic analysis of the Global Burden of Disease Study 2019 showed that the incidence of CRC in China is significantly high when adjusted for age.^3^ Despite recent progress in detecting and treating early CRC, long‐term survival rates for advanced CRC are still low. Therefore, there is a critical need to explore effective therapies for patients with advanced CRC. In 2015, Le et al. first reported that immune checkpoint inhibitors (ICIs) provide significant survival benefits for patients with deficient DNA mismatch repair (dMMR) metastatic CRC,^4^ marking the start of a new era of immunotherapy for CRC. Recently, Wang et al. showed that combining anti‐PD‐1, HDAC inhibitor and anti‐VEGF may be a promising treatment regimen for patients with unresectable chemotherapy‐refractory locally advanced or metastatic microsatellite stable/proficient mismatch repair (MSS/pMMR) colorectal cancer.^5^ The introduction of ICIs into the treatment of mCRC with dMMR has transformed the therapeutic landscape by extending patient survival.^6^ The expression level of programmed death ligand‐1 (PD‐L1) is a crucial factor in determining the effectiveness of PD‐1/PD‐L1 blockade therapy.^7^ N‐linked glycosylation enhances the stability of the PD‐L1 protein, which is vital for its interaction with PD‐1. Furthermore, this interaction promotes immune evasion, suggesting that PD‐L1 glycosylation could be a viable target for optimising cancer immunotherapy.^8^, ^9^, ^10^

Cancer‐associated fibroblasts (CAFs) are the major stromal cells in the tumour microenvironment (TME) and play crucial roles in cancer initiation, progression, metastasis, as well as in metabolism, angiogenesis, immunity and therapeutic resistance.^11^, ^12^, ^13^ The advancement of single‐cell RNA sequencing (scRNA‐seq) technology has allowed researchers to uncover the heterogeneity of CAFs in different cancer types. Notably, some CAFs exhibit tumour‐suppressive functions.^14^, ^15^ The vast majority of CAFs blunt the clinical efficacy of immunotherapy, making them an emerging target for anticancer immunotherapy. The regulatory pathways related to PD‐1/PD‐L1 inhibitor immunotherapy that are influenced by CAFs vary across different solid tumours.^14^, ^16^, ^17^, ^18^ CAFs can increase PD‐L1 expression on cancer cells by deriving various cytokines and vesicles, which help tumours evade the immune response.^14^ However, whether CAFs can induce the upregulated expression of N‐linked glycosylation PD‐L1 via specific glycosyltransferases is still unanswered.

Herein, we identify that CAFs increase PD‐L1 glycosylation through EDEM3, facilitating immune evasion. Additionally, high‐EDEM3 expression predominantly recruits M2‐like macrophages, creating a barrier‐protective tumour microenvironment that underpins PD‐1/PD‐L1 blockade resistance in vivo. By analysing the Cancer Genome Atlas (TCGA) CRC dataset, metabolomics data and flow cytometry, we clarify that EDEM3 predominantly activates the recruited M2‐like macrophages via a glucose metabolism‐dependent mechanism. Blocking glucose utilisation antagonises the recruiting and polarising M2‐like macrophages synergistically with PD‐1 antibody to improve anticancer immunity. Our study proposes a potential strategy to improve checkpoint blockade therapy by restricting glucose to disrupt EDEM3‐induced M2‐like macrophage trafficking.

## 2 RESULTS

### 2.1 CAFs upregulated PD‐L1 glycosylation and increased its expression on tumour cell membranes via glycosyltransferase EDEM3

**FIGURE 1:**
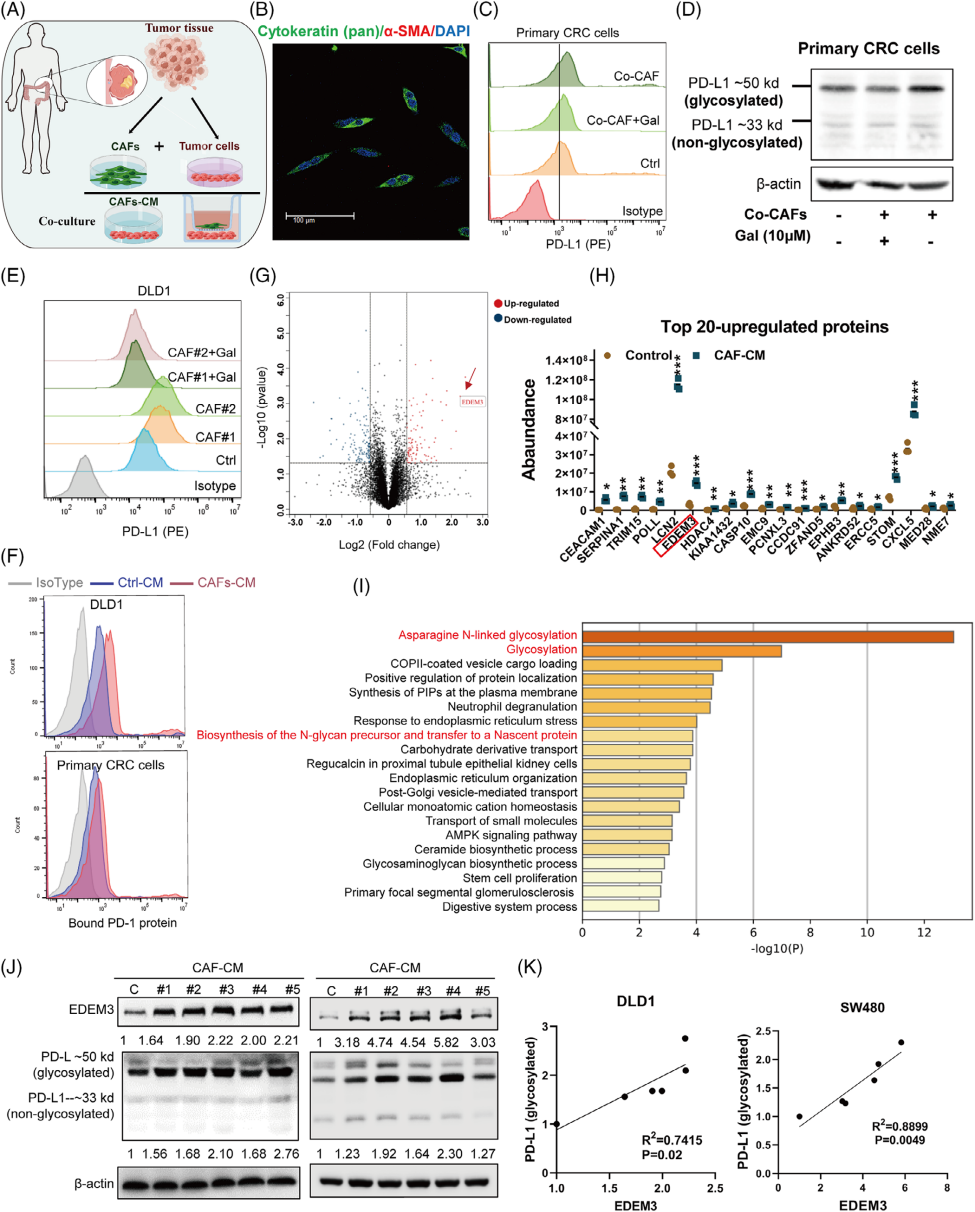
CAFs upregulated the PD‐L1 glycosylation protein expression via glycosyltransferase EDEM3. (A) Schema of the coculture model. (B) Immunofluorescence staining of cytokeratin (pan) and α‐SMA in primary CRC cells isolated from colon cancer tissues. (C) Flow cytometry analysis of cell surface PD‐L1 levels in primary CRC cells upon indicated treatment for 48 h. (D) Immunoblotting analysis of PD‐L1 glycosylation and non‐glycosylation protein levels in primary CRC cells. (E) Flow cytometry analysis of cell surface PD‐L1 levels in DLD1 cells upon indicated treatment for 48 h (*n* = 2 independent assays). (F) Flow cytometry assays of the binding of PD‐1 to PD‐L1 in CRC cells upon treatment with CAFs‐CM for 48 h (*n* = 2 independent assays). CAFs‐CM was collected from CAFs #2. Primary CRC cells and CAFs #2 were acquired from the same patient. (G) Volcano plots showing differential protein expression (fold change ≥ 1.5; FDR < 0.05) between Control medium and CAFs‐CM‐treated DLD1 cells (data from three biological replicates). Red dots: proteins significantly upregulated; blue dots: proteins significantly downregulated. (H) Scatter dot plot for the top 20 differentially expressed proteins by quantitative proteomic analysis. Student's *t*‐test. (I) Bar graph illustrating the enriched GO terms associated with the EDEM3 in COAD tumour analysed based on Metascape. (J) Immunoblotting analysis of PD‐L1 glycosylation and EDEM3 protein levels in DLD1 (left) and SW480 (right) cells upon treatment with CAFs‐CM. (K) Correlation analysis of EDEM3 and PD‐L1 glycosylation expression. Pearson's correlation test. All data are shown as mean ± SD; *p < 0.05, **p < 0.01, ***p < 0.001.

Previous studies have demonstrated that CAFs enhance PD‐L1 expression in tumour cells,^19^, ^20^ and highlighted the significance of PD‐L1 glycosylation in immune suppression.^8^, ^9^, ^21^ We asked whether human colorectal cancer‐derived CAFs affected PD‐L1 glycosylation of CRC cells. We established patient‐derived primary CRC cells/CAF coculture systems by adding patient‐derived CAFs (Figure 1A,B). The PD‐L1 protein on the tumour cell membrane increased following coculture (Figure 1C). Consistently, the upregulation of PD‐L1 N‐linked glycosylation was confirmed by immunoblotting (Figure 1D). After coculture with CAFs for 48 h, N‐glycosylation of PD‐L1 increased in CRC cells, resulting in a significant increase in surface PD‐L1 levels (Figure 1E and Figure S1A–C). Galunisertib, a TGF‐β inhibitor known to inhibit CAF activation,^22^ can block the effect of CAFs on PD‐L1 glycosylation (Figure 1C–E and Figure S1A,B). Similar to the coculture system, CAF‐derived conditioned medium (CAF‐CM) shared the ability to upregulate N‐glycosylation of PD‐L1 (Figure S1D,E). Primary CAFs can only be cultured for six to seven passages, limiting their use in extensive and duplicate assays; therefore, we opted to use CAF‐CM for subsequent studies, as PD‐L1 functions only when localised to the cell membrane and interacts with PD‐1. To determine whether CAFs‐induced PD‐L1 glycosylation affects CRC cell immunosuppression, we compared the immunosuppression activity in vitro. CRC cells, including cell line DLD1 and primary cells with CAFs‐CM incubation, exhibited more PD‐1 protein binding to the cell surface than cells with a control medium (Figure 1F). Therefore, the results suggest that activated CAFs significantly enhance PD‐L1 glycosylation and facilitate immune evasion. However, how CAFs induce PD‐L1 glycosylation in CRC cells remains unreported. Next, we asked whether CAFs modify PD‐L1 glycosylation through glycosyltransferase.

Using label‐free quantitative proteomics, we conducted a comprehensive study to obtain a detailed view of the effect of CAFs‐CM on DLD1 cells in the PD‐L1 glycosylation regulatory process. The result showed that there were 100 proteins upregulated and 143 proteins downregulated in DLD1 cells after CAFs‐CM treatment (fold change [FC] ≥ 1.5, *p*‐value < 0.05) (Figure 1G and Table S1). The most significantly changed proteins were highly involved in metabolic pathways (Figure S2A). Among the top hits was a glycosyltransferase, EDEM3 (Figure 1G,H), an enzyme known for its role in N‐glycan trimming and quality control in the endoplasmic reticulum.^23^, ^24^ To investigate the biological significance of EDEM3 in CRC cancer, we performed gene ontology (GO) enrichment analysis using TCGA databases. GO analysis revealed that EDEM3‐related genes were predominantly enriched in ‘Asparagine N‐linked glycosylation’ (R‐HAS‐446203) and ‘Glycosylation’ (GO:0070085) categories (Figure 1I). Additional immunoblotting analyses showed a significant positive correlation between EDEM3 protein expression and PD‐L1 glycosylation in CRC cells treated with CAF‐CM (Figure 1J,K). This correlation was similarly confirmed in six CRC cell lines (Figure S2B,C). These data suggested that the glycosyltransferase EDEM3 may regulate PD‐L1 glycosylation in CRC cells, providing a confident understanding of the regulatory process.

### 2.2 EDEM3 promotes immune evasion by increasing PD‐L1 glycosylation and cell surface PD‐L1

**FIGURE 2:**
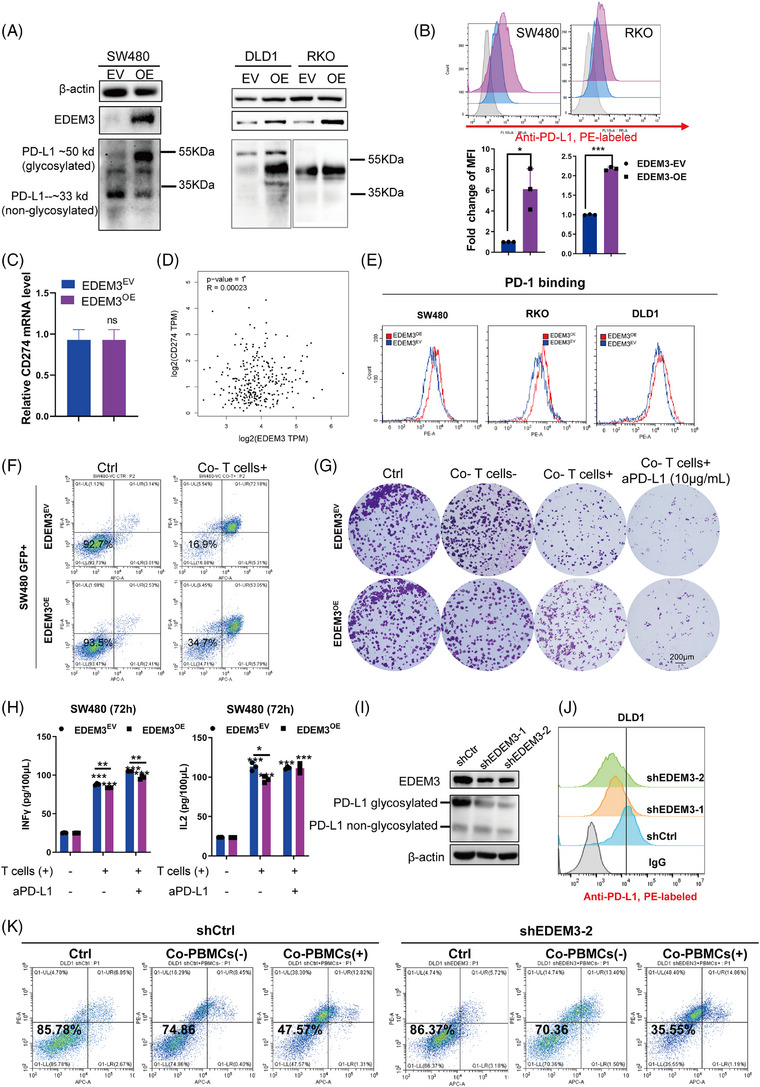
EDEM3 upregulates PD‐L1 glycosylation, increases cell‐surface PD‐L1 and limits T cell‐dependent antitumour immunity. (A) Western blot confirmed the stable overexpression of EDEM3 by lentiviral transduction in SW480, DLD1 and RKO cells. EV: control vector, OE: over‐expression vector. (B) Flow cytometry (upper panel) analysis of cell surface PD‐L1 levels in EDEM3^OE^ cells. The PD‐L1 MFI was quantified (*n* = 3 independent assays, unpaired Student's *t*‐test). MFI: mean fluorescence intensity. (C) Relative PD‐L1 mRNA expression levels were measured with RT‐qPCR (*n* = 3), unpaired Student's *t*‐test. (D) EDEM3 and PD‐L1 (CD274) mRNA expression correlation from 270 colon cancer tumours (COAD) from TCGA. Pearson's correlation test. (E) Flow cytometry assays of the binding of PD‐1 to PD‐L1 in indicated cells (*n* = 2 independent assays). (F) Cell apoptosis (GFP^+^) upon coculture with activated T cells was determined by flow cytometry. EDEM3^EV^ and EDEM3^OE^ cells were cotransfected with GFP; consequently, apoptosis was assessed in the GFP^+^ cell populations. Percentages of cell survival are shown. (G) The survival cells after treatment under the indicated conditions are shown by crystal violet staining. Representative of two experiments are shown. T cells (−) means T cells were not activated, and T cells (+) were activated by CD3/28. (H) Interferon (IFN)‐gamma and interleukin (IL)‐2 cytokine expression was measured by ELISA. *n* = 2 independent assays, obtained from different T cells isolated from two health donors, each arising from three quantification replicates. Unpaired Student's *t*‐test. ***p < 0.001 vs. corresponding control, ‘_’ means comparison between these two groups. (I) Immunoblotting analysis of PD‐L1 glycosylation in DLD1 shEDEM3 cells. (J) Flow cytometry analysis of cell surface PD‐L1 levels in DLD1 shEDEM3 cells. (K) Cell apoptosis upon coculture with activated T cells was determined by annexin‐V/PI assay. The values within each panel indicate % of survival cells. PBMCs (−) means PBMC cells were not activated, and PBMCs (+) means PBMC cells were activated by CD3/28. All data are shown as mean ± SD; *p < 0.05, **p < 0.01, ***p < 0.001.

We wondered whether EDEM3 could upregulate N‐linked glycosylated PD‐L1 expression; thus, EDEM3 stable overexpression CRC cell lines were constructed by lentiviral vector transfection. Both in MSI and MSS CRC cells, EDEM3 overexpression increased glycosylated PD‐L1 levels (Figure 2A). Additionally, the increased expression of cell‐surface PD‐L1 in EDEM3‐overexpressing cells was confirmed by flow cytometry assays (Figure 2B). Cell proliferation was determined using the IncuCyte Zoom (Live Content Imaging); no apparent acceleration was observed in EDEM3^OE^ cells (Figure S3A). Notably, PD‐L1 (CD274) mRNA levels were not affected in EDEM3^OE^ cells (Figure 2C and Figure S3B), and no correlations between CD274 and EDEM3 mRNA levels were found in RNA‐seq datasets from TCGA database (Figure 2D), indicating that EDEM3 regulates PD‐L1 expression by protein post‐translational modification. Interestingly, the global analysis of protein N‐glycosylation in EDEM3^OE^ cells did not vary significantly across the whole PVDF membranes, except for specific bands of certain molecular weights (Figure S4), implying specific regulation of glycosylated modifications. To test whether the upregulated PD‐L1 glycosylation mediated by EDEM3 affects PD‐1 binding, we incubated the EDEM3^OE^ CRC cells with human PD‐1 Fc chimera protein. Flow cytometry assay showed that PD‐1 binding to EDEM3^OE^ cells was significantly increased (Figure 2E and Figure S3C). Together, these results indicate that EDEM3 mediates glycosylation, increases the total and surface levels of PD‐L1, and enhances the binding ability of PD‐1.

Subsequently, coculture with EDEM3^OE^ CRC cells significantly inhibited T‐cell‐mediated tumour killing as indicated by an increased percentage of viable cells (Figure 2F,G and Figure S3D–G) and attenuated IFN‐γ and IL‐2 secretion in the corresponding samples (Figure 2H and Figure S3H). Next, we added PD‐L1 neutralising antibodies to the coculture system to identify whether EDEM3‐mediated PD‐L1 operates in T‐cell suppression. Blocking tumour‐surface PD‐L1 with neutralising antibodies increased the sensitivity of EDEM3^OE^ cells to T‐cell‐mediated killing and cytokine secretion (Figure 2G,H and Figure S3G,H). Conversely, we confirmed that EDEM3 knockdown decreased PD‐L1 expression both at the glycosylation protein levels and on the cell membranes (Figure 2I,J). Coculturing of activated cells (Co‐PBMCs^+^) and EDEM3 knockdown DLD1 cells also promoted a modest increase in tumour cell killing (Figure 2K). The glycosyltransferase EDEM3 can upregulate PD‐L1 glycosylation, promote cell‐surface PD‐L1 expression, and limit the antitumour activity of cytotoxic T lymphocytes (CTLs) response in vitro.

### 2.3 EDEM3 accelerates PD‐1 antibody treatment resistance by recruiting M2‐like macrophages

**FIGURE 3:**
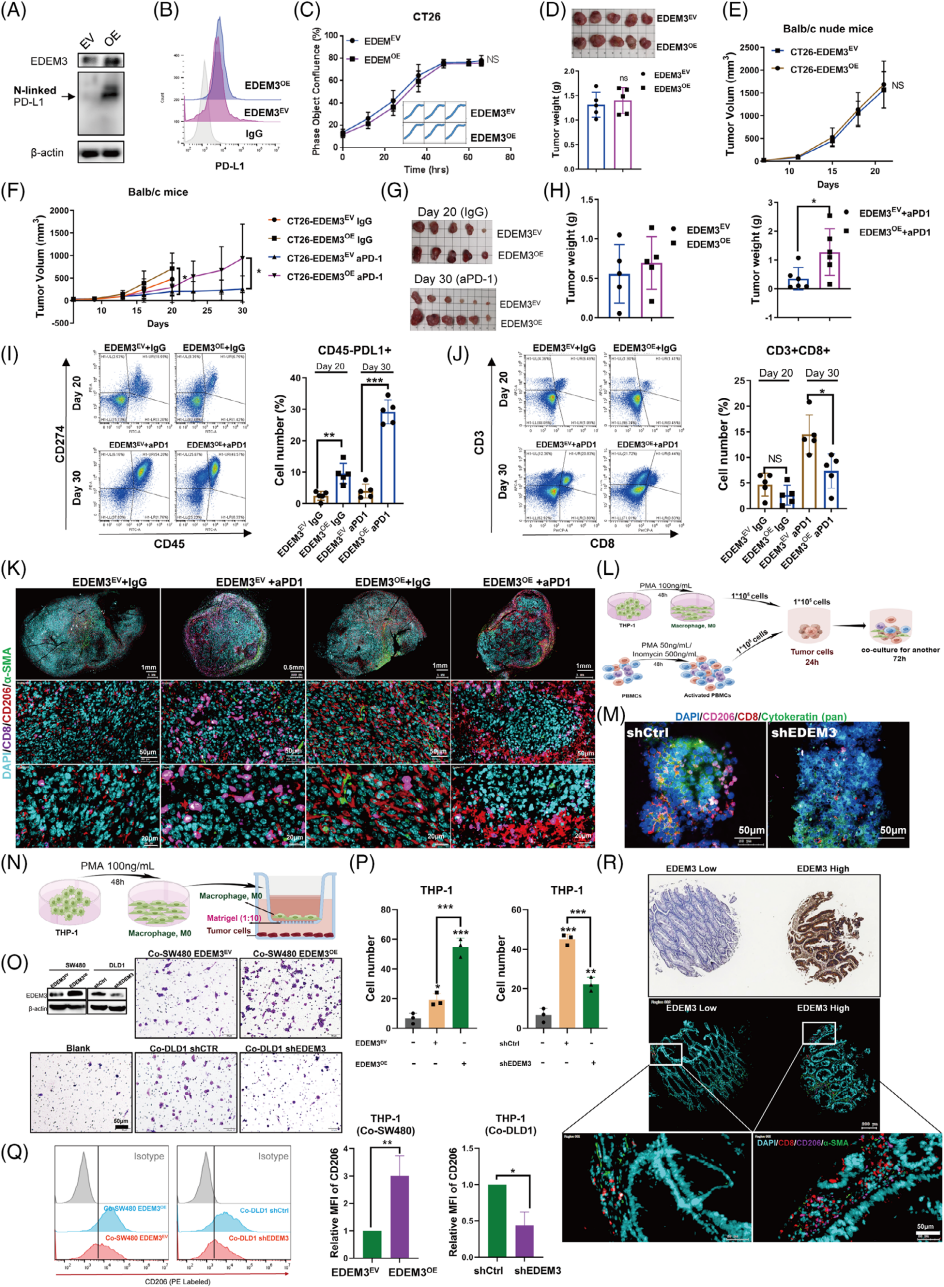
High EDEM3 expression confers resistance to αPD‐1 treatment. (A) Stable overexpression of EDEM3 by lentiviral transduction in CT26 cells was confirmed by Western blot. EV: control vector, OE: overexpression vector. (B) Flow cytometry (upper panel) analysis of cell surface PD‐L1 levels in CT26 EDEM3^OE^ cells. (C) Growth curves for the indicated cells were measured by the IncuCyte ZOOM live‐cell imager (*n* = 3). Two‐way ANOVA. (D and E) The indicated subcutaneous tumour images (D, upper panel), tumour weight (D, lower panel) and tumour growth curves (E) in immunodeficient mice are shown (*n* = 5). (F–H) Tumour growth curves (F), tumour images (G) and tumour weight (H) of mice bearing CT26 EDEM3^OE^ or EDEM3^VC^ tumours following treatment with IgG or PD‐1 antibody (*n* = 5 or 6 mice per group). (I) The proportion of PD‐L1^+^ cells within the CD45^−^ population of in vivo tumours is shown by flow cytometry. *n* = 5, Unpaired *t*‐test. (J) The proportion of CD3^+^CD8^+^ cells was analysed by flow cytometry in the indicated tumours. *n* = 5, Unpaired *t*‐test. (K) Tumour tissues were immunostained by multiplex IHC (mIHC) with anti‐CD8, CD206 and α‐SMA as indicated. (L) Schematic workflow of the 3D coculture system. (M) mIHC images of tri‐culture spheroids (DLD1+THP‐1+PBMCs). (N) Schematic showing the CRC cells cocultured with human monocytic leukaemia cells (THP‐1) stimulated by PMA. (O) The invasion assays towards the indicated CRC cells cocultured with macrophages for 24 h. (P) Quantification of invasion assay by counting the number of invaded cells/field. *n* = 3, One‐way ANOVA. (Q) The expression of CD206 (M2 marker) in THP1 after the indicted treatment was analysed and quantified by flow cytometry. (R) High and low EDEM3‐expressed tumour tissues were immunostained by mIHC with anti‐CD8, CD206 and α‐SMA as indicated. All data are shown as mean ± SD; *p < 0.05, **p < 0.01, ***p < 0.001.

We next examined the effect of EDEM3 on tumour growth and the therapeutic efficacy of PD‐1 blockade in vivo. As expected, there was a significantly increased glycosylated and cell surface level of PD‐L1 in EDEM^OE^ CT26 cells, but no effect on cell spreading (Figure 3A–C). To further explore the effect of EDEM3 on the tumour‐immune response of CRC in vivo, a mouse model of EDEM3 overexpression (EDEM3^OE^) subcutaneous CT26 tumours was developed. EDEM3‐overexpressing CT26 cells were implanted subcutaneously into immunodeficient mice, and tumour growth was monitored. Consistently, tumours derived from EDEM^OE^ CT26 cells were not rapidly proliferating or increasing in size (Figure 3D,E). To evaluate whether EDEM3 could direct tumour immune escape and affect tumour growth, we compared the growth of EDEM3^EV^ and EDEM^OE^ tumours in BALB/c mice. Although not statistically significant, we observed a trend towards EDEM3‐overexpressed being involved in tumour promotion after control IgG treatment (Figure 3F–H). Most remarkably, EDEM^OE^ tumours were completely resistant to the effects of PD‐1 blockade after an initial transient response to PD‐1 inhibition (Figure 3F–H). Reinfeld et al. showed CD45^−^ cells were predominantly cancer cells in subcutaneous tumours.^25^ Furthermore, we detected an increase in PD‐L1 protein level in the membrane fraction (CD45^−^/PD‐L1^+^) in the EDEM3^OE^ tumour compared to control tumours, particularly the tumours after PD‐1 antibody treatment by flow cytometry (Figure 3I and Figure S5A). Correspondingly, the infiltration of CD8^+^ T cells (CD3^+^/CD8^+^) was also significantly reduced (Figure 3J and Figure S5B). These observations differ significantly from previous reports on tumour PD‐L1 expression and are now approved as a predictive biomarker for PD‐L1 blockade. This has prompted us to delve deeper into the underlying cause of this discrepancy, adding a new dimension to the field.

Tumours can evade the immune system by creating an immunosuppressive tumour microenvironment (TME).^26^ In addition to CAFs, accumulating evidence indicates that M2‐like macrophages, also known as tumour‐associated macrophages (TAMs), play a critical role in promoting antitumour immune responses.^27^, ^28^ Our findings provide strong evidence for this, as we observed a significant increase in CD206^+^ macrophages surrounding highly resistant EDEM3^OE^ tumours (Figure 3K). CD206^+^ macrophages were observed surrounding the viable tumour foci, and this phenomenon was consistently noted across individual EDEM3^OE^ tumours from different mice after anti‐PD‐1 treatment (Figure S6A). These macrophages are known to form the functional tumour barrier, limiting T‐cell entry and activity, thus aiding in the evasion of destruction by cytotoxic T cells.

We used a 3D coculture system in vitro to validate the association between EDEM3 expression and immune cell infiltration, as phorbol‐12‐myristate‐13‐acetate (PMA)‐treated THP‐1 cells induce the monocytic cell line to differentiate into monocyte‐derived macrophages (Figure 3L). The data revealed fewer CD206^+^ macrophages (purple) surrounding CRC spheroids with low EDEM3 expression (green), while the number of CD8^+^ T cells (red) increased in these cultures (Figure 3M). We conducted a migration assay using PMA‐treated THP‐1 cells to verify whether EDEM3 affects the chemotactic migration of macrophages (Figure 3N). Coculturing with the EDEM3‐overexpressing CRC cells significantly enhanced the invasive ability of macrophages (Figure 3O,P). Conversely, the PMA‐treated THP‐1 cells showed a significantly reduced invasive ability when cocultured with the shEDEM3 cells (Figure 3O,P). Macrophages cocultured with EDEM3‐high CRC cells (SW480 EDEM3^OE^ and DLD1 shCtrl) exhibited a high expression of CD206 (an M2 marker) resembling the M2‐like TAMs phenotype. In contrast, those cocultured with EDEM3‐low CRC cells (SW480 EDEM3^EV^ and DLD1 shEDEM3) did not show this phenotype (Figure 3Q). However, coculturing did not significantly alter the expression of CD86 (an M1 marker) (Figure S6B). Finally, we examined the expression levels of EDEM3, CD8^+^ T cells, M2‐like TAMs and CAFs in human CRC tumour samples. As shown in Figure 3R, EDEM3‐high was associated with a large part of M2 macrophage subpopulations that block killing induced by CD8^+^ T cells. Collectively, these data suggest that EDEM3 confers resistance to PD‐1 blockade by promoting macrophage migration and M2‐like polarisation, further enhancing tumour cell survival by suppressing immune responses by CD8^+^ T cells.

### 2.4 EDEM3 expression correlates with immunogenicity and the response to immunotherapy in colorectal cancer

**FIGURE 4:**
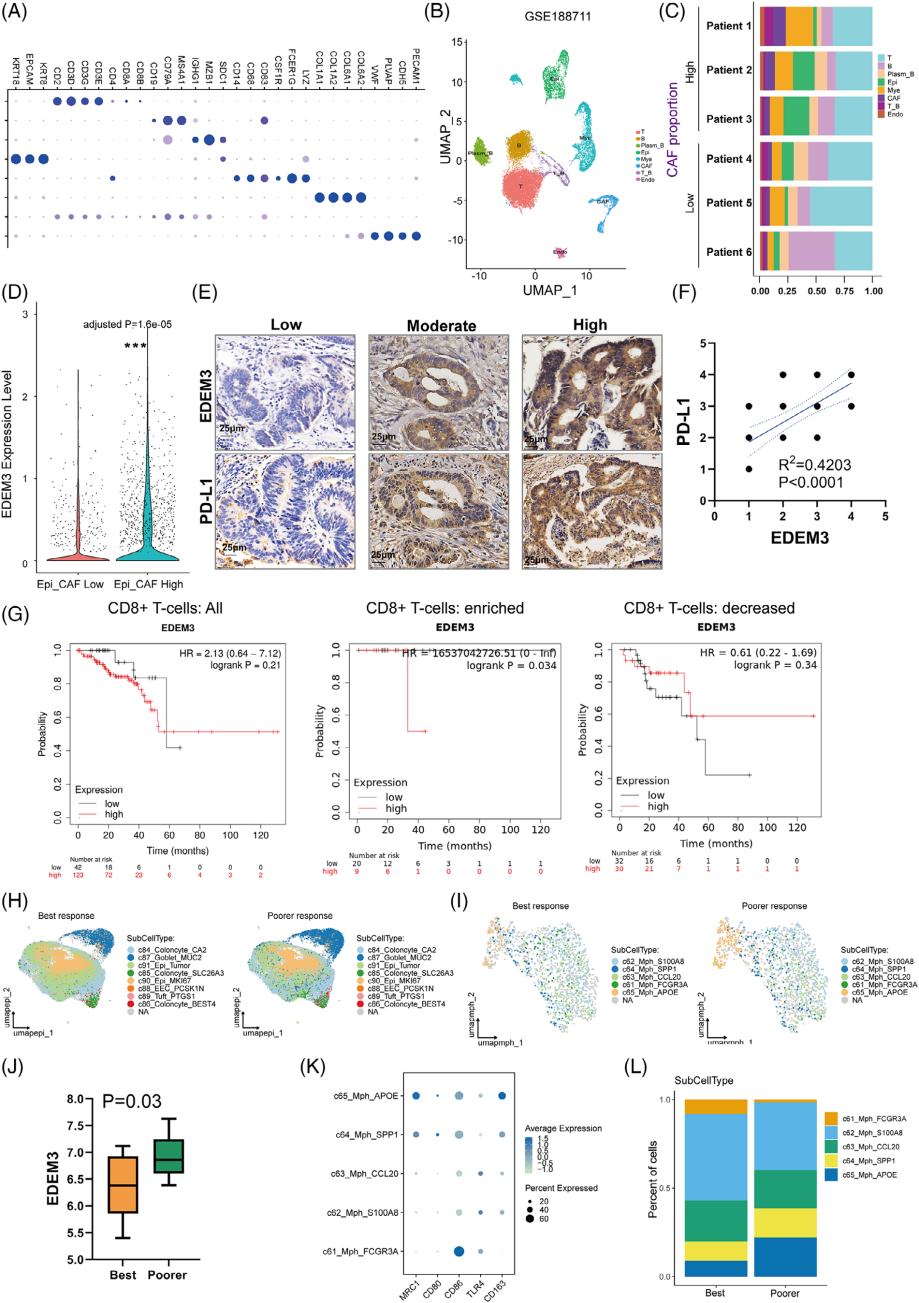
EDEM3 expression correlates with immunogenicity and response to immunotherapy in colorectal cancer. (A) The indicated cell signatures were derived from known markers. Epi: epithelial cells, Mye: myeloid cells, Endo: endothelial cells. (B) Uniform manifold approximation and projection (UMAP) plot of annotated cell types. (C) The fraction of cells that originated from each of the six patients. (D) The EDEM3 mRNA level of epithelial cells in tumours with high and low CAF infiltration. The data (A–D) were derived from the GEO database (GSE188711). (E) IHC staining of EDEM3 and PD‐L1 is shown. (F) Relative immunohistochemical scores of EDEM3 and PD‐L1 are shown (*n* = 35). Some of the dots on the graphs overlap and represent more than one specimen. (G) Kaplan–Meier overall survival curves of CRC (*n* = 165) patients with high and low EDEM3 mRNA expression obtained from the online Kaplan–Meier plotter database (http://kmplot.com/analysis) (left). Overall survival of CRC patients with enriched (*n* = 29) (middle) or decreased (*n* = 62) (right) CD8^+^ T cells and with high or low expression of EDEM3. (H and I) UMAP plots showing epithelial cell (H) and macrophage clusters (I), with different colours representing different subtypes. (J) The expression of EDEM3 between the best response group and the poor response group in the tumour cells. The Wilcoxon rank‐sum test was used to assess the differences. (K) Dot plot showing the mean expression of associated gene signatures in each macrophage subtype. (L) The proportion of each macrophage subtype in the best and the poor response groups. The data (H–L) were derived from the GEO database (GSE236581). Statistical analyses were performed by the two‐tailed log‐rank test. All data are shown as mean ± SD; ***p < 0.001.

Next, we analysed the expression of EDEM3 in human CRC tissues compared to normal tissues, finding that EDEM3 exhibited significantly higher expression in CRC tissues (Figure S7A). Then, we obtained in vivo evidence showing EDEM3 expression in CRC tumour cells associated with CAF infiltration, using a single‐cell RNA sequencing database. We found that the EDEM3 expression in epithelial cells (marked by KRT18, EPCAM, KRT8) was significantly higher in tumour tissues with a large proportion of CAFs (marked by COL1A1, COL1A2, COL6A1, COL6A2) (Figure 4A–D) in the GSE188711 CRC dataset.^24^ Subsequently, we confirmed a significant positive correlation between EDEM3 and PD‐L1 expression by serial section and IHC staining (Figure 4E,F). We then analysed the associations of EDEM3 expression with patient outcomes based on the RNA‐seq datasets using the Kaplan–Meier plotter (http://kmplot.com/analysis). As shown in Figure 4G, high EDEM3 expression correlated with poor prognosis only in the CD8^+^ T‐cell‐enriched population, not in the CD8^+^ T‐cell‐depleted or overall populations in READ. This phenomenon was also confirmed in breast, ovarian and pancreatic cancers (Figure S7B–D). These data suggest that CAFs‐induced EDEM3 is important in promoting PD‐L1 expression during tumour progression by suppressing antitumour immune responses.

Then, we asked whether high‐EDEM3 expression in tumour cells would affect the response to immunotherapy by analysing CRC patient responses to the PD‐1 antibody using the Gene Expression Omnibus (GSE236581). *Poorer response* was defined as the totality of stable disease and partial responses (*n* = 10), and complete responses were further graded into the best response (*n* = 12). First, unsupervised clustering combined with canonical marker‐based annotation revealed epithelial cell type and macrophage (Mph) cell type, according to the report by Chen et al.^29^ (Figure 4H,I). Consistent with our finding, we verified that the tumours with upregulated EDEM3 have a poorer response to PD‐1 blockade therapy (Figure 4J). SPP1^+^ Mph and APOE^+^ Mph clusters represent a resident‐like macrophage subpopulation with an M2 phenotype (Figure 4K), as evidenced by the production of anti‐inflammatory cytokines and pro‐tumourigenesis properties.^30^, ^31^ Specifically, we revealed that the less‐SPP1^+^ Mph and APOE^+^ Mph cells in patients with low EDEM3‐expressed tumours was associated with the best response (Figure 4L). These observations demonstrate that CAF‐mediated‐EDEM3 may promote M2‐like polarisation and enhance M2‐like macrophage‐dependent functions to inhibit immunotherapy response.

### 2.5 Blocking glucose utilisation decreases PD‐L1 N‐glycosylation and attenuates EDEM3‐induced recruitment and M2‐like polarisation of macrophages

**FIGURE 5:**
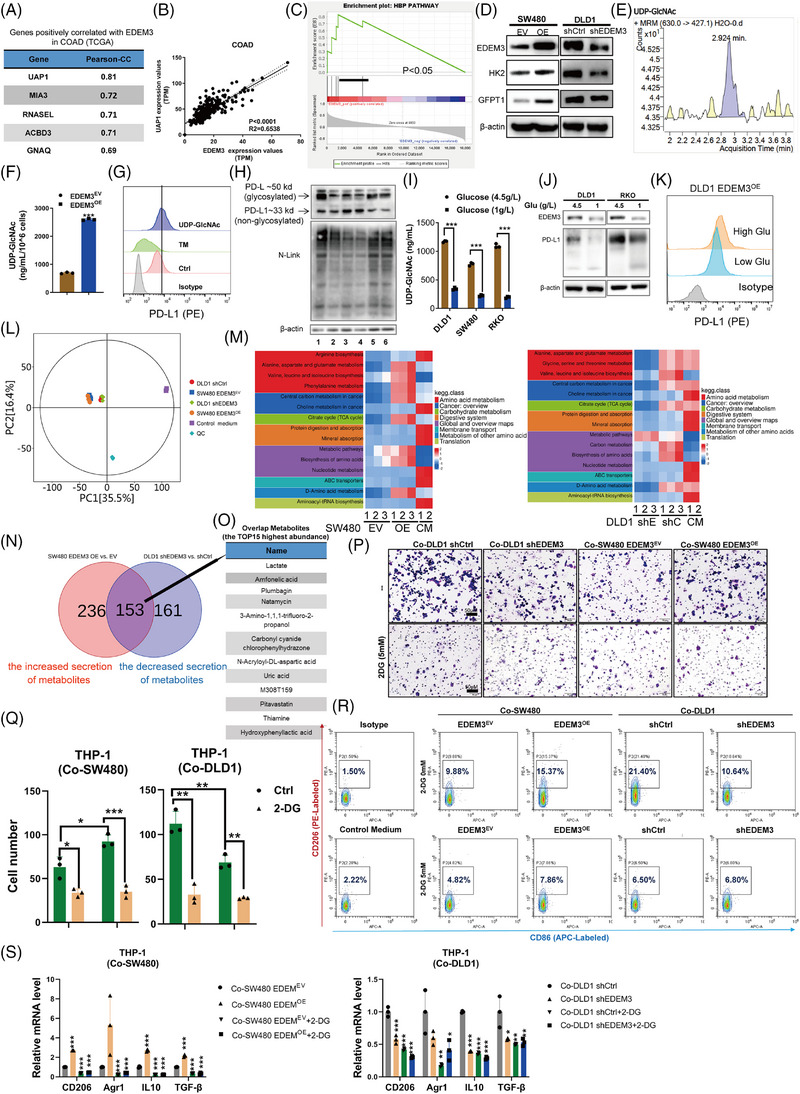
Blockade of glucose utilisation decreases PD‐L1 N‐glycosylation and reverses EDEM3‐induced recruitment and M2‐like polarisation of macrophages. (A and B) Correlations were performed by Spearman correlation test based on the TCGA databases. Pearson's correlation test. (C) GSEA analysis of EDEM3 impacts on HBP pathway. The genes in the HBP pathways, HK2, GFAT, UAP1, GNPNAT1 and PGM3, are chosen. (D) Immunoblotting analysis of EDEM3, HK2 and GFPT1 in indicated cells. (E) Establishment of the UDP‐GlcNAc expression profiling method by LC‐MS/MS. (F) UDP‐GlcNAc concentrations measured by LC‐MS/MS (*n* = 3). Unpaired Student's *t*‐test. (G) Flow cytometric analysis of PD‐L1 expression on cell surface after the indicated treatment. TM: tunicamycin, 1 µg/mL. (H) Immunoblotting analysis of global N‐glycosylation and PD‐L1 glycosylation levels in RKO cells treated with medium containing different levels of glucose (Glu) and glutamine (Gln) or 2‐DG (5 mM) for 48 h. Line 1: C (normal medium), Line 2: C+5 mM 2‐DG, Line 3: 0.5 g/L Glu+1 mM Gln, Line 4: 0.5 g/L Glu+2 mM Gln, Line 5: 4.5 g/L Glu+1 mM Gln, Line 6: 4.5 g/L Glu+2 mM Gln. (I) UDP‐GlcNAc concentrations measured by LC‐MS/MS after the indicated treatment (*n* = 3), Unpaired Student's *t*‐test. (J) Immunoblotting analysis of PD‐L1 glycosylation and EDEM3 levels in DLD1 cells treated with different glucose (Glu) levels for 48 h. (K) Flow cytometric analysis of PD‐L1 expression on EDEM3^OE^ cell surface after the indicated treatment. (L) PCA plots were made using the differentially expressed metabolites (*n* = 3). (M) The heatmap shows enriched KEGG metabolic pathways with *q*‐values ≤0.05. (N) Overlapping analysis of metabolites identified by untargeted metabolomics. (O) The overlap secretion of metabolites from the top 15 highest abundance from these two cell lines. (P) Invasion assays of PMA‐stimulated THP‐1 cells after coculturing with indicated CRC cells for 24 h. (Q) Quantification of the matrigel invasion assay by counting the number of invaded cells/field (*n* = 3). (R) FACS analysis determined CD86^+^ M1 and CD206^+^ M2 macrophages. (S) Expression of markers associated with M2 polarisation in THP‐1 of the indicated groups (*n* = 3). All data are shown as mean ± SD; *p < 0.05, **p < 0.01, ***p < 0.001.

To understand the modified glycosylation mechanism by EDEM3, we used Pearson correlation analysis in colorectal cancers (COAD and READ) based on the TCGA databases. The hexosamine‐biosynthesis pathway (HBP), a subbranch of glucose metabolism, plays a crucial role in carcinogenesis.^29^ Uridine diphosphate N‐acetylglucosamine (UDP‐GlcNAc), the end product of HBP, serves as a donor substrate for both O‐GlcNAcylation and O‐ and N‐linked glycosylation.^30^ UDP‐N‐acetylglucosamine pyrophosphorylase 1 (UAP1), the last enzyme of HBP, can increase 10‐fold levels of UDP‐GlcNAc.^31^ Our analysis revealed that UAP1 has the strongest positive correlation with COAD and READ among the examined genes (Figures 5A,B and S8A,B). Gene set enrichment analysis (GSEA) demonstrated a strong correlation between EDEM3 and the HBP pathways (Figure 5C). Then, we confirmed that high EDEM3‐expressing cells showed upregulation of the key pathway proteins hexokinase 2 (HK2) and glutamine‐fructose‐6‐phosphate transaminase 1 (GFPT1), which are the rate‐limiting enzymes of glycolysis and the HBP (Figure 5D). Besides, we also confirmed this phenomenon in the EDEM3^OE^ tumours (Figures S8C and S9). GFAT1 facilitates the HBP metabolic flow, leading to production of the end product UDP‐GlcNAc. Indeed, in parallel with high levels of EDEM3 expression, a high content of UDP‐GlcNAc was found (Figures 5E,F and S8D). Supplementing with exogenous UDP‐GlcNAc also increased PD‐L1 expression on the cell membrane, as demonstrated by flow cytometry analysis (Figures 5G and S8E). Glucose and glutamine are metabolised through HBP to produce UDP‐GlcNAc.^32^ Therefore, we performed experiments to analyse the global protein N‐glycosylation and PD‐L1 glycosylation in cells treated with varying concentrations of glucose and glutamine for 48 h. Then, the data showed that low glucose, without glutamine, or treatment with 2‐deoxy‐D‐glucose (2‐DG), a competitive inhibitor of glucose metabolism, reduced global N‐glycosylation and PD‐L1 glycosylation in RKO cells (Figures 5H and S8F). Consistently, the concentration of UDP‐GlcNAc, as measured by LC‐MS/MS, was significantly reduced (Figure 5I). Additionally, PD‐L1 glycosylation decreased along with the markedly reduced levels of EDEM3 protein in a low‐glucose medium (1 g/L) (Figure 5J). Our previous study reported that CAFs enhance glucose uptake and metabolism by increasing fluidity of cell membranes (Figure S10A).^11^ We then observed that glucose uptake decreased in cells treated with sodium palmitate (PA) due to reduced membrane fluidity (Figure S10B), which reversed the CAFs‐CM‐induced PD‐L1 expression on the cell surface (Figure S10C,D). To test the specificity of the glucose effect, we showed that under normal culture conditions (4.5 g/L glucose), CAFs‐CM significantly upregulated EDEM3 expression. This effect was abolished under low glucose conditions (0.5 g/L), suggesting CAFs increased EDEM3 expression in CRC cells as a result of enhanced glucose uptake (Figure S10E,F). Glucose deprivation undoubtedly led to a significant decrease in PD‐L1 levels on the cell membrane, which are upregulated by EDEM3 (Figure 5K). These data suggest that glucose limitation attenuates PD‐L1 glycosylation mediated by EDEM3. This finding underscores the importance of glucose metabolism in regulating PD‐L1 glycosylation and its potential as a therapeutic target.

Our untargeted metabolomics analysis of the supernatant deepened our comprehension of metabolite changes. Principal component analysis revealed a clear pattern of metabolite changes across different cell lines (Figure 5L). The KEGG pathway enrichment analysis heatmap showed that the most consistently altered pathway sets occurred in high‐EDEM3‐expression cells (SW480 EDEM3^OE^/DLD1 shCtrl) (Figure 5M). We then identified potential factors by overlapping the secretion of metabolites. A set of 153 secretion of metabolites was regulated by EDEM3 in both DLD1 and SW480 cells (Figure 5N). Of these metabolites, lactate was the most abundant, serving as a key product of aerobic glycolysis (Figure 5O). Colegio et al. demonstrated that tumour‐derived lactic acid plays an important role in the polarisation of TAMs.^32^ We next asked whether the glycolytic inhibitor impairs the recruitment and polarisation of M2‐like macrophages. As expected, we found that inhibiting glucose utilisation with 2‐DG in the EDEM3 high‐expression cells significantly suppressed the invasion of PMA‐treated THP‐1 cells (Figure 5P,Q). Consistent with the changes in chemotactic migration, PMA‐stimulated THP‐1 cells exhibited decreased expression of M2 markers (CD206‐positive) after coculturing with 2‐DG‐treated EDEM3‐high CRC cells (Figure 5R). At the same time, the ratio of M1‐type (CD86 positive) macrophages was not influenced (Figure 5R). In particular, the increased expression of M2 markers, Arg1, CD206, TGF‐β and IL‐10 in PMA‐treated THP‐1 when cocultured with high‐EDEM3 CRC cells could be reversed by 2‐DG (Figure 5S). Our results indicate that EDEM3‐driven glucose metabolism plays a crucial role in upregulating PD‐L1 glycosylation and recruiting M2‐like macrophages. Therefore, blocking glucose utilisation may hold potential therapeutic implications, offering hope for future treatments.

### 2.6 FMD synergising with 2‐DG improves the efficacy of anti‐PD‐1 therapy by increasing cytotoxic CD8^+^ T cells and diminishing macrophage infiltration

**FIGURE 6:**
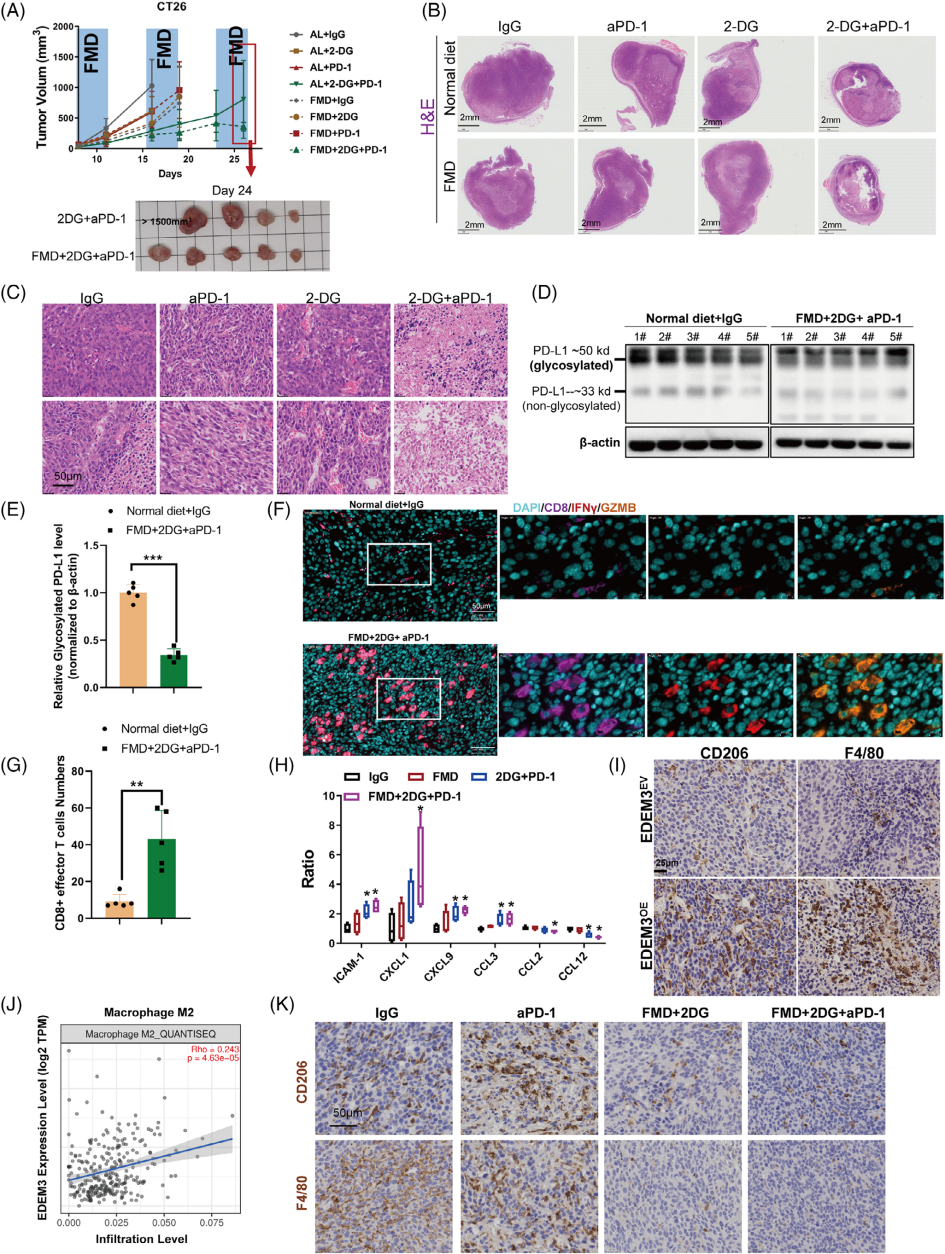
FMD plus 2‐DG enhances the efficacy of anti‐PD‐1 treatment by PD‐L1 deglycosylation. (A) CT26 tumour growth in immunocompetent BALB/c syngeneic mice treated with isotype control, 2‐DG and anti‐PD‐1, and fed with standard diet or FMD (*n* = 5). (B and C) H&E staining of tumour tissue sections after the indicated treatment. Representative of the whole tumour sections (B) and local magnified view (C). (D) Detection of glycosylated PD‐L1 in indicated treated tumour masses (*n* = 5 per group). The uncropped original image is provided in Figure S8. (E) Relative glycosylated PD‐L1 protein levels were quantified. The relative gray value of protein = protein of interest/internal reference. (F) The IFN‐γ^+^/GZMA^+^/CD8^+^ T cells were detected by multiplex immunofluorescence imaging. (G) IFN‐γ^+^/GZMA^+^/CD8^+^ T‐cell numbers were counted after the indicated treatment from the random fields of view at 20× magnification, preferred to avoid regions of tumour necrosis. (H) Cytokine profiles (containing 40 cytokines) carried out on tumour homogenates from mice after the indicated treatment was analysed using RayBio Mouse Cytokine Antibody Array, One‐way ANOVA. (I) Immunohistochemistry images of tumours showing M2 macrophage infiltration cells in different treatment groups. (J) The association between EDEM3 mRNA level and M2 macrophage was evaluated by the TIMER 2.0 database. (K) Immunohistochemistry images of tumours showing M2 macrophage infiltration cells in different treatment groups. All data are shown as mean ± SD; *p < 0.05, **p < 0.01, ***p < 0.001.

Based on the above findings, we hypothesised that decreased glucose supply and utilisation could disrupt M2‐like macrophage trafficking by EDEM3 overexpression, thereby enhancing anti‐PD‐1 immunotherapy in vivo. FMD alters various growth factors and metabolites, producing environments that can reduce cancer cells’ ability to adapt and survive, thus improving the efficacy of cancer therapies. Hence, BALB/c mice with a subcutaneous CT26 tumour were subjected to one or three cycles of a 4‐day FMD or treated with 2‐DG, anti‐PD‐1, alone or in combination. The results showed that neither anti‐PD‐1 monotherapy nor 2‐DG alone significantly improved antitumour efficacy in either the standard diet group (ad libitum, AL) or the FMD group compared to the IgG‐only group (Figure 6A). However, anti‐PD‐1 treatment combined with 2‐DG effectively inhibited tumour growth in mice on both a standard diet and FMD, particularly in those on the FMD (Figure 6A). H&E staining of tumour sections showed extensive cell death in anti‐PD‐1+2‐DG‐treated tumours, regardless of whether the mice were on a standard diet or FMD, compared to the control groups (Figure 6B,C). Notably, the levels of glycosylated PD‐L1 were substantially reduced in the tumour masses of mouse models treated with FMD+2‐DG+anti‐PD‐1 (Figure 6D,E and Figure S11A,B).

We also confirmed that fasting decreases PD‐L1 glycosylation and surface expression in vitro (Figure S11C,D). This finding corresponded with the increased tumour‐infiltrating CD8^+^ T cells and augmented CD8^+^ T‐cell responses, as evidenced by high IFN‐γ and granzyme B expression in CD8^+^ T cells (Figure 6F,G). To demonstrate whether blocking glucose utilisation via the cytokines secreted reverses the EDEM3‐induced increase in M2 macrophages and decreased CD8^+^ T‐cell infiltration, cytokine profiles (containing 40 cytokines) of tumour tissues were analysed using the RayBio Mouse Cytokine Antibody Array. Interestingly, cytokines that positively regulate CD8^+^ T‐cell activation and promote tumour infiltration and cytotoxicity of CD8^+^ effector T cells, such as ICAM1,^33^ CXCL9^34^ and CCL3,^35^ were increased in the FMD+2‐DG+anti‐PD‐1 group. In contrast, the levels of M2 macrophages chemokines CCL2 and CCL12^36^, ^37^ were diminished (Figure 6H). Our previous study confirmed that depleting M2 macrophages could augment CD8^+^ T‐cell infiltration, effectively inhibiting tumour growth and improving responses to immunotherapy.^38^ Recent studies indicate that resistance to PD‐1/PD‐L1 antibody is caused by limited T‐cell infiltration and massive suppressive tumour‐associated macrophages.^39^ To validate the association between M2 macrophage infiltration and immunotherapy resistance in EDEM3^OE^ tumours, we demonstrated that EDEM3^OE^ tumours displayed significant markers of substantial M2 macrophage infiltration (Figure 6I). Furthermore, TIMER2.0 analysis revealed the substantial infiltration of M2 macrophages in EDEM3 high‐expression CRC tumours (Figure 6J). Besides the increased infiltrating CD8^+^ T cells, we demonstrated that combining 2‐DG with either anti‐PD‐1 or an AL or FMD diet significantly reduced M2 macrophage infiltration (Figure 6K). Based on these results, we confirmed our hypothesis that blocking glucose metabolism by the FMD+2‐DG enhances immunotherapy effectiveness via increasing CD8^+^ cytotoxic T cells and reducing M2 macrophage infiltration.

### 2.7 Glycosylated enzyme EDEM3 overexpression exacerbates anti‐PD‐L1 resistance, which can be reversed upon glucose deprivation

**FIGURE 7:**
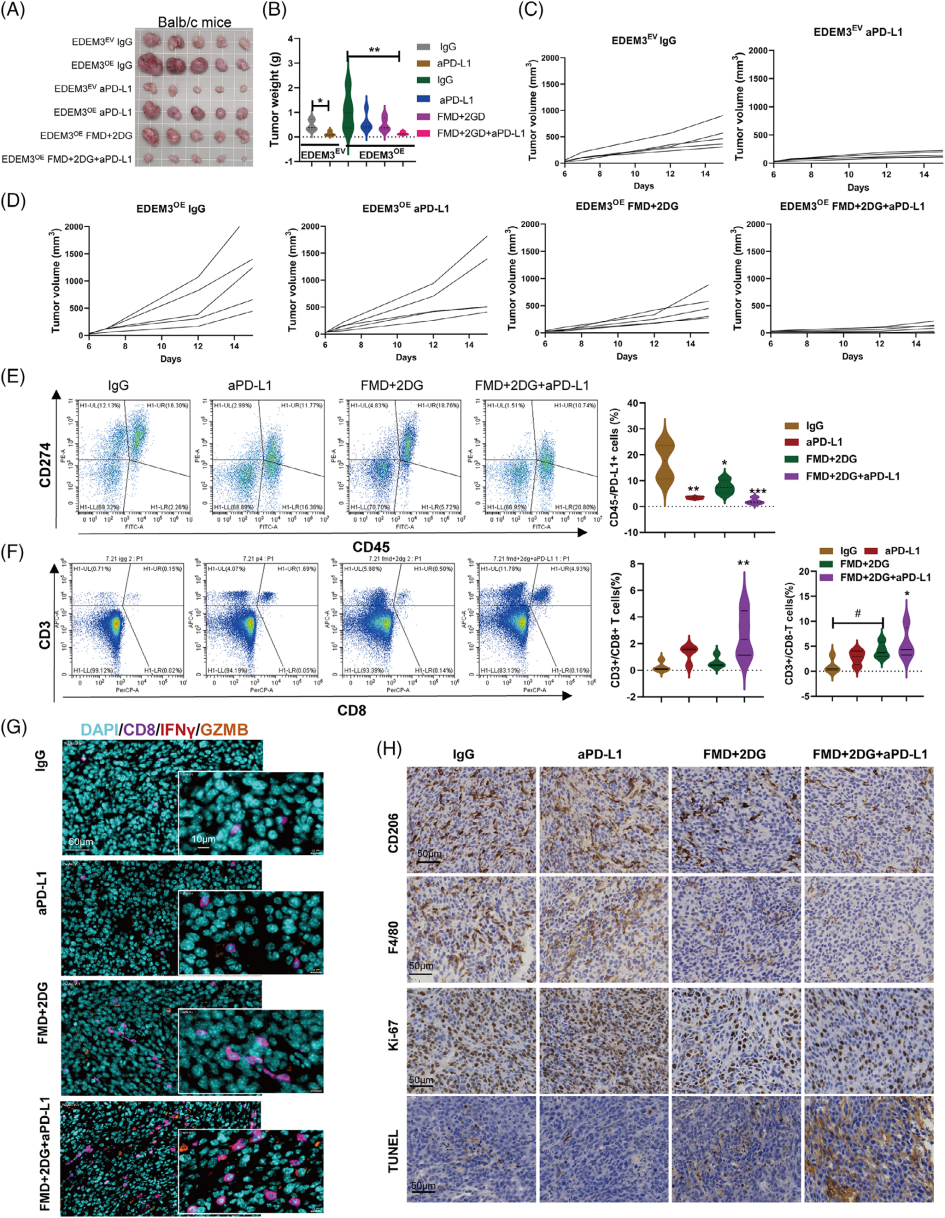
Glucose restriction produces an enhanced antitumour effect and ablates PD‐1/PD‐L1 blockade resistance in EDEM3 high‐expression tumours. BALB/c mice were injected s.c. with 1 × 10^6^ CT26 EDEM3^OE^ or EDEM3^VC^ cells. Six days after tumour injection, mice were treated i.p. with 10 mg/kg anti‐PD‐L1 antibodies or IgG, FMD+1 g/kg of 2‐DG, or a combination of anti‐PD‐L1 or IgG and FMD+2‐DG. (A–D) The graph of CT26 tumour growth is shown. Tumour images (A), tumour weight (B) and tumour growth curve (C and D), *n*  =  5. (E) The proportion of PD‐L1^+^ cells within the CD45^−^ population was analysed by flow cytometry in the indicated tumours. One‐way ANOVA. (F) The proportion of CD3^+^CD8^+^ cells was analysed by flow cytometry in the indicated tumours. One‐way ANOVA. (G) Intracellular IFN‐γ and GZMB‐producing CD8 T cells in the indicated tumours were detected by the multiplex immunofluorescence imaging. (H) Immunohistochemical results of CD206, F4/80, Ki67 and TUNEL expression in the respective group are shown. All data are shown as mean ± SD; *p < 0.05, **p < 0.01, ***p < 0.001.

To explore effective strategies for EDEM3 overexpression in CRC therapy that can reverse resistance and lower toxicity, we used CT26 cells to express empty vector (EDEM^EV^) or harbour EDEM3 overexpression (EDEM3^OE^) in the BALB/c mice. After 6 days of implanting these two cell lines into syngeneic mice, the mice received the corresponding drug treatments. As shown in Figure 7A–D, the tumours in the EDEM3‐overexpression group were larger in both volume and weight compared to the EV group following IgG treatment. Although this difference was not statistically significant, there was a trend towards significance. In addition, EDEM3 overexpression in CT26 tumour cells did not significantly inhibit tumour growth in the presence of the PD‐L1 antibody treatment (Figure 7A–D and Figure S12A,B), likely because the basal PD‐L1 expression was already high (Figure 3I). Then, the flow cytometry analysis compared tumours treated with IgG to those treated with FMD plus 2‐DG. As expected, inhibiting glucose metabolism in vivo resulted in a lower tumour cell‐intrinsic surface level of PD‐L1 in EDEM3^OE^ tumours, as CD45^−^ were predominantly cancer cells^25^ (Figure 7E). However, the decreased expression of CD274 on the tumour cell surface in the anti‐PD‐L1‐treated group was due to the effective blocking of the PD‐L1 antibody. Consistent with the reduced tumour growth rate, we also observed a striking increase in the infiltration of CD3^+^/CD8^+^ T cells (Figure 7F). This finding was further verified by multiplex immunofluorescence staining (Figure 7G). Meanwhile, the intracellular cytokines staining confirmed the enhanced cytotoxicity of CD8^+^ T cells (Figure 7G). Again, there was less M2‐like macrophage infiltration in FMD combined with 2‐DG plus anti‐PD‐L1‐treated tumours (Figure 7H, upper panel). Additionally, this treatment exhibited anti‐colon cancer activity, as indicated by lower Ki67 expression (Figure 7H, middle panel) and significantly enhanced cytotoxic effect, reflected by increased TUNEL positivity (Figure 7H, lower panel). Anti‐PD‐L1 treatment alone suppressed proliferation and enhanced apoptosis of CT26 EDEM3^EV^ tumour cells in mice (Figure S12C). Validation of the data support that glucose metabolism repression can reverse EDEM3 overexpression‐mediated resistance to the PD‐1/PD‐L1 blockade and promote antitumour immunity in vivo.

### 2.8 Combination of FMD and 2‐DG boosts the effectiveness of anti‐PD‐L1 therapy in a colon cancer model with low EDEM3 expression

**FIGURE 8:**
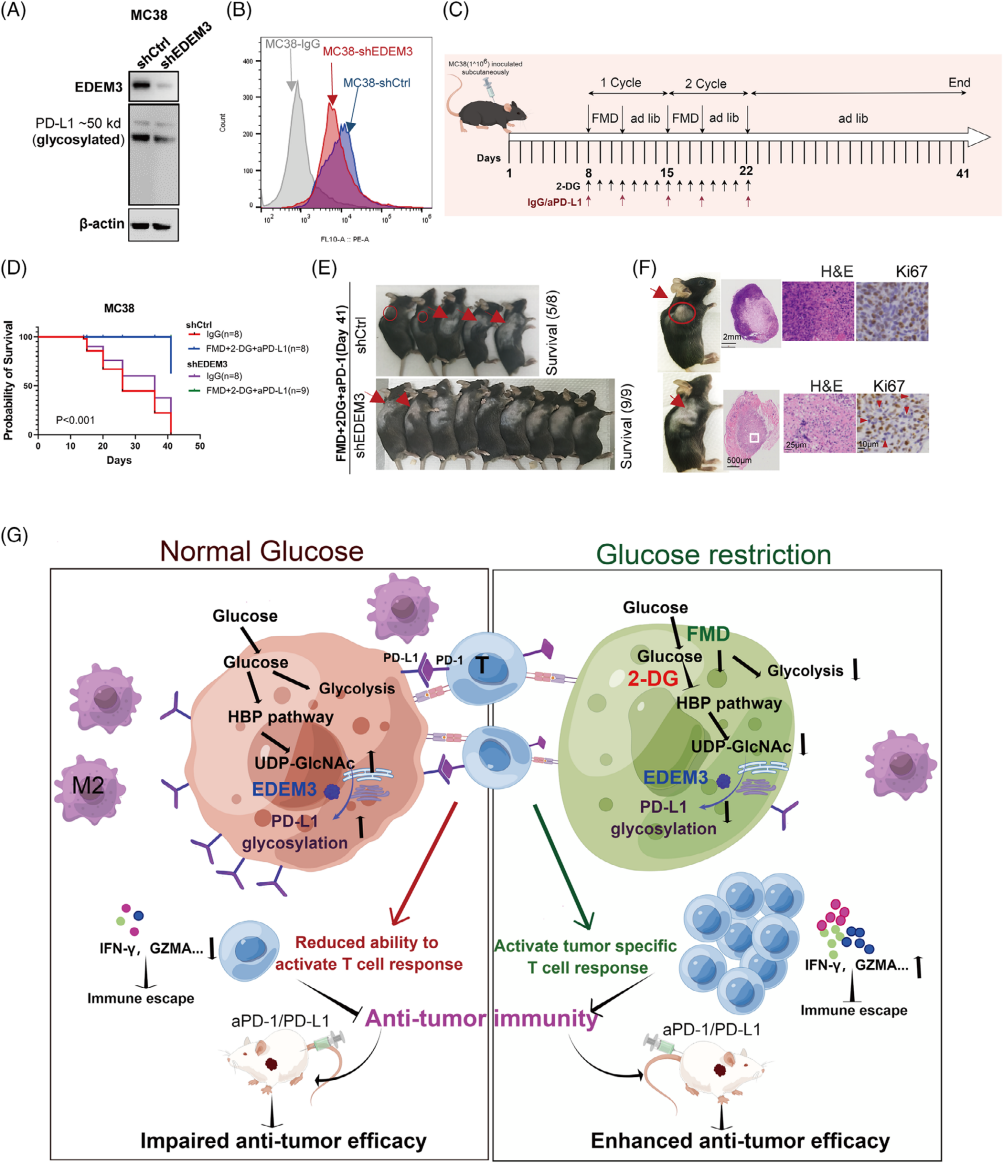
FMD combined with 2‐DG augments antitumour immunity, leading to tumour regression in EDEM3‐knockdown tumours. (A) Western blot analysis to compare EDEM3 expression and PD‐L1 glycosylation in control and EDEM3‐knockdown MC38 cells. (B) Flow cytometry analysis of the cell surface levels of PD‐L1 expression on MC38 shEDEM3 cells. (C) Schedule of tumour implantation and the treatment schedule of MC38 subcutaneous tumour models. (D) Survival of mice bearing syngeneic MC38 shCtrl or shEDEM3 tumours following the indicated treatment (*n* = 8 or 9 mice per group). Two‐sided log‐rank survival test. (E) Mice with a subcutaneous shEDEM3 cell xenograft tumour after the indicated treatment. (F) H&E staining and Ki67 expression in the respective group. (G) Schematic of the mechanism of action of removing EDEM3‐induced PD‐L1 glycosylation and decreasing CD206^+^ M2‐like macrophages by glucose restriction enhancing antitumour immunity.

As shown in Figure 8A,B, we confirmed in vitro that EDEM3 knockdown reduces PD‐L1 glycosylation and cell membrane expression levels in MC38 cells. To test if the effect of glucose metabolism suppression on anti‐PD‐L1 immunotherapy could be replicated in another mouse model, we used C57BL/6 mice with subcutaneous MC38 shCtrl or shEDEM3 tumours (Figure 8C). There was no statistically significant difference in survival between the shCtrl and shEDEM3 tumours; however, knocking down EDEM3 led to a slight delay in tumour growth (Figure 8D). This suggested that EDEM3 is involved in tumour immunity during progression, supported by a significant correlation between EDEM3 expression and poor prognosis in the CD8^+^ T‐cell‐enriched population (Figure 4G, Figure S7B–D). The results indicated that FMD+2‐DG+anti‐PD‐L1 treatment significantly slowed tumour growth and prolonged survival, suggesting that inhibiting glucose utilisation by tumour cells boosts anticancer immunity (Figure 8D). At the terminal time, five out of eight mice in the MC38‐shCtrl group survived (Figure 8D,E). Remarkably, all nine mice treated with FMD+2‐DG+anti‐PD‐L1 survived the challenge (Figure 8D,E). Furthermore, all tumours in the MC38‐shEDEM3 group exhibited nearly complete regression, making the regression site barely visible (Figure 8E). H&E staining indicated that the shEDEM3 tumour treated with FMD+2‐DG+anti‐PD‐L1 had a mixed histological structure, very few residual tumour cells remained in the tumour regression bed (Figure 8F, middle panel). Additionally, sections were stained for Ki67, a proliferation marker, revealing that tumour cells with lower EDEM3 expression post treatment with FMD+2‐DG+anti‐PD‐L1 were rarely Ki67‐positive (Figure 8F, right panel). Our data present glucose metabolism as a novel actionable target, and its loss‐of‐function sensitises tumours to T‐cell‐induced apoptosis in immunotherapy, opening up a promising avenue for further research and potential therapeutic development (Figure 8G).

## 3 DISCUSSION

The PD‐1/PD‐L1 axis is crucial for immune escape and immunotherapy. Importantly, translated N‐linked glycosylation of PD‐L1 enters the cell membrane and binds to PD‐1, rendering T cells anergic and leading to T‐cell exhaustion and dysfunction. New regulators of PD‐L1 are emerging that control its levels on the cell surface, potentially enhancing therapeutic efficacy.^8^, ^9^, ^10^, ^40^ As key components in the tumour microenvironment, in addition to promoting malignant growth and invasion, CAFs mediate immune escape. Galbo et al. showed that specific CAF subtypes are associated with resistance to anti‐PD1 or anti‐PD‐L1 immunotherapy in the metastatic bladder, melanoma and kidney cancer.^18^ CAFs can mediate immune escape via upregulating PD‐L1 expression.^14^, ^19^, ^40^, ^41^, ^42^ However, whether CAFs upregulate PD‐L1 is connected to glycosylation via glycosyltransferase remains ill‐defined.

This study highlights the essential role of CAFs in facilitating PD‐L1 glycosylation through the glycosyltransferase EDEM3. However, high expression levels of EDEM3 promote M2‐like polarisation and chemotactic migration of macrophages, leading to PD‐1/PD‐L1 blockade resistance. A bioinformatic analysis performed by Guo et al. showed that PD‐L1 is positively associated with immunosuppressive macrophages, and cytokines derived from these cells contribute to their polarisation.^43^ Similarly, the most recent report by Murai et al. identified that PD‐L1 is upregulated in HCC cells, accompanied by significant infiltration of CAFs and M2 macrophages.^44^

Heavily glycosylated PD‐L1, a glycosylated cell‐surface receptor protein, stabilises itself and promotes tumour immune escape.^8^, ^9^, ^40^ Conversely, targeting PD‐L1 glycosylation would significantly improve antitumour immune responses.^9^, ^45^, ^46^ We demonstrated that EDEM3 increases glucose metabolism by upregulating HK2 and GFPT1 expression, while also increasing UDP‐GlcNAc levels. The HBP pathway promotes tumour growth and mediates tumour immunoresistance.^47^ In line with our metabolomics data, we found significant correlations between EDEM3 and lactate levels, confirming increased glucose utilisation activity. As a byproduct of glycolysis, lactic acid produced by tumour cells has a critical function in the M2‐like polarisation of macrophages and emerges as a prominent immunosuppressive metabolite.^32^, ^48^ Thus, it seemed reasonable that glucose deprivation can decrease UDP‐GlcNAc production and reduce PD‐L1 glycosylation. Meanwhile, we observed a significant increase in tumour‐infiltrated CD8^+^ T cells and a reduction in M2‐like macrophages following the FMD+2‐DG+aPD‐L1 treatment. Cortellino et al. showed that FMD can increase the efficacy of immunotherapy by expanding early exhausted effector T cells.^49^ Guo et al. provided a proof of concept to eliminate tumour immune evasion for improved cancer treatment with combined HK2 inhibitors and immune checkpoint blockade therapies.^50^ Shao et al. demonstrated that 2‐DG can reverse the immunosuppression caused by PARP inhibitors in triple‐negative breast cancer by deglycosylating PD‐L1.^51^ Ketogenic diets that significantly lower glucose levels in vivo, when combined with the anti‐CTLA‐4 antibody, markedly retarded tumour progression by decreasing PD‐L1 expression and promoting antitumour immunity pathways.^46^ The latest reports confirmed that inhibition of Glut1 genetically and pharmacologically sensitises tumours to antitumour immunity and synergises with anti‐PD‐1 therapy through the TNF‐a pathway.^52^ In our study, we also confirm the role of glucose metabolism restriction in reversing the increased cell‐surface levels of PD‐L1 and the resistance to PD‐1/PD‐L1 inhibitor therapy caused by EDEM3 overexpression.

Our findings not only uncover a critical mechanism driving the upregulation of PD‐L1 glycosylation expression by CAFs via glycosyltransferase EDEM3 and enhance M2‐like macrophage infiltration and confer resistance to ICBs in CRC cells, but also inspire potential solutions. This research supports developing therapeutic strategies that could reverse PD‐L1 glycosylation and reduce M2‐like tumour‐associated macrophages infiltration by limiting glucose utilisation. The strategy could serve as a powerful tool against EDEM3‐induced ICI‐resistant CRC cancer, highlighting the urgency and relevance of our research in the pursuit of innovative cancer treatments.

## 4 MATERIALS AND METHODS

Cell culture and transfection, CAF primary cultures, label‐free proteomic analysis, plasmids, immunohistochemistry, cell viability and apoptosis assay, PD‐L1 and PD‐1 binding assay, detection of cell surface PD‐L1, quantification of cellular UDP‐GlcNAc concentrations, T‐cell subsets sorting, Western blot analysis, multiplex immunofluorescence staining, primary human T cells are isolated for coculture experiments and assay for T‐cell‐mediated cytotoxicity, animal diets, animal models and drug treatments, statistical analysis and study approval are provided in Supporting Information.

## AUTHOR CONTRIBUTIONS

Conceptualisation: **Xiaoxia Liu** and **Yanxin Luo**. Methodology: **Xiaoxia Liu**; **Shaoyong Peng**; **Minshan Wu**; **Gaopo Xu** and **Guannan Tang**. Investigation: **Zixu Yuan**; **Jingrong Weng**; **Jinxin Lin**; **Yumo Xie**; **Liangliang Bai** and **Xiaoxia Liang**. Writing—original draft: **Xiaoxia Liu** and **Qian Yan**. Writing—review and editing: **Yanxin Luo** and **Xiaoxia Liu**. Funding acquisition: **Xiaoxia Liu**; **Huichuan Yu** and **Yanxin Luo**. Resources: **Yanxin Luo** and **Huichuan Yu**. Supervision: **Xiaolin Wang**; **Meijin Huang** and **Yanxin Luo**.

## CONFLICT OF INTEREST STATEMENT

The authors declare they have no potential conflicts of interest.

## ETHICS STATEMENT

All animal experiments complied with the ARRIVE guidelines and were operated according to protocols approved by the Institutional Laboratory Animal Care and Use Committee of The Sixth Affiliated Hospital, Sun Yat‐sen University, China (IACUC‐ 2023041701). Ethical approval was gained from the Institutional Review Board of the Sixth Affiliated Hospital of Sun Yat‐sen University, China (2024ZSLYEC‐403).

## Supporting information



Supporting Information

Supporting Information

## Data Availability

All data supporting the findings of this study are available with the article, or from the corresponding author upon reasonable request.
