# Supplementary material for: Disrupting EDEM3‐induced M2‐like macrophage trafficking by glucose restriction overcomes resistance to PD‐1/PD‐L1 blockade
Source: Clin Transl Med. 2025 Jan 3;15(1):e70161. doi: 10.1002/ctm2.70161 (PMC11702414; doi:10.1002/ctm2.70161)
Supplement: Supplementary file 1 — Supporting Information [file CTM2-15-e70161-s001.pdf]

Supplementary Materials and Methods

Cell Culture and Transfection

RKO (CRL-2577™, RRID: CVCL_0504), DLD1 (CRL-221™, RRID:CVCL_0248), SW480 (CCL-228™, RRID:CVCL_0546) and CT26 (CRL-2638™, RRID:CVCL_7254) cells were purchased from the American Type Culture Collection (Manassas, VA, USA). The murine colon cancer cell line MC38 (RRID: CVCL_B288) was purchased from the National Infrastructure of Cell Line Resource (Beijing, China). SW480 cell lines were cultured in Dulbecco's Modified Eagle Medium (DMEM) (Corning, Cat. #: 10-013-CV) or RPMI 1640 medium (Corning, Cat. #: 10-040-CV) supplemented with 10% FBS (Corning, Cat. #: 35-081-CV) and 1% penicillin/streptomycin (Corning, Cat. #: 30-002-CI). Cells were maintained in a humidified, 5% CO2 atmosphere at 37 °C. All cells were tested for mycoplasma contamination every two months, and all tests were negative.

Primary Human CRC cells and CAFs culture process as described previously[1]. To generate conditioned media, CAFs were seeded into 10 cm dishes and allowed to reach 90% confluence. Media was replaced with DMEM (4.5g/L glucose) supplemented with pen/strep and 10% FBS and cells were incubated for 48hr. Media was collected, filtered with a 0.8mm filter to remove cell debris and then frozen at -80 ℃. For CAF-CM + Gal collection, CAFs were treated with10 μM Galunisertib for 48hr, and then

was generated by incubating DMEM supplemented with pen/strep and 10% FBS for 24hr. Control CM was generated under the same conditions in a dish without cells.

Label-free proteomic analysis

DLD1 (1.5×10^5) cells were seeded in triplicate in 6-well plates overnight at 37°C. CAF-CM (25%) or control CM (25%) and complete DMEM (75%) were added to each well for 48 h incubation. Then, the cells were collected. The proteomic experiment was performed and data analysis was supported by Wayen Biotechnologies Co., Ltd. (Shanghai, China).

Plasmids

A lentiviral plasmid was generated containing full-length EDEM3 by recombining the vector, pEZ-Lv201, and the empty vector control was purchased from GeneCopoeia™ (Shanghai, China). Sequences of specific shRNAs used in this study were: human shEDEM3-1:GCTGATGAACTCATGCCTTTA; human shEDEM3-2:GGATGACAGTAACTTCGATTG; Human EDEM3 gene silencing was performed by lentiviral transduction, pLKO.1-CMV-copGFP-PURO; mouse EDEM3 gene silencing was performed by lentiviral vector, pLKO.1-TRC-copGFP-2A-PURO, and the corresponding negative control were obtained from Tsingke Biotechnology Co., Ltd. (Beijing, China).

T cell subsets sorting

Ct26 tumor masses were collected and washed by PBS three times. Then

tumors were cut into 1 mm^3-thick tissue block, adding 6 ml of dissociation solution [RPMI1640 medium supplemented with 10% FBS, collagenase type IV (1mg/mL) (Sigma-Aldrich, Cat#: C5138) and DNase I (10 μg/mL) (Sigma-Aldrich, Cat#: DN25)] for 50 min at 37 ◦C. Cells were passed through 70-μm cell strainers, washed, and resuspended in staining buffer (Biolegend, Cat#: 420201). Cell suspensions were stained using the Fixable Viability Dye (1 μL/mL) (eBioscience, Cat#: 65-0865-14) to enable the exclusion of dead cells and were surface stained with tube A: anti-CD8a (Biolegend, Cat#: 100731), anti-CD3 (Biolegend, Cat#:100235); tube B: anti-CD45 (Biolegend, Cat#:103107), anti-PD-L1 (Biolegend, Cat #:124308), 20 minutes for surface staining. All labeled cells were detected using a Beckman flow cytometry system and analyzed with CytExpert.

Flow cytometry for THP-1

The following fluorescent dye-labeled antibodies purchased from eBioscience were used in this study: anti-Hu CD86 (APC) (Cat#2660380) and anti-Hu CD206 (PE) (Cat# 2647760). The indicated cells were collected, stained with the cell surface marker (CD86) for 30 mins on ice, then fixed/permeabilized using a fixation/permeabilization kit (eBioscience). Add the CD206 antibody and incubate in the dark at room temperature for 30 mins. Cell pellets were resuspended in PBS with 2% FBS for flow cytometry analysis. All labeled cells were detected using a

Beckman flow cytometry system and analyzed with CytExpert.

Multiplex fluorescent immunohistochemical staining (mIHC)

The as-prepared tumor sections were stained according to the instructions of the four-color multiplex fluorescence immunohistochemical staining kit (Absin, Cat#:abs50012) and blocked with goat serum (ZSGB-BIO, Cat#:ZLI-9056) before incubation with antibodies. The antibodies involved in experiment include CD8 (Cell Signaling Technology, Cat#: 98941, diluted at 1:100), IFNγ (Affinity Biosciences, Cat# DF6045, diluted at 1:100), GZMB (Affinity Biosciences, Cat# AF0175, diluted at 1:100), aSMA (Proteintech, Cat#67735-1-IG, diluted at 1:200), CD206 (Cell Signaling Technology, Cat#24595, diluted at 1:200) The nuclei were stained with DAPI before sealing, and all sections were scanned by a fluorescent scanning camera (TissueFAXS Plus-S, TissueGnostics).

Primary human T cells are isolated for coculture experiments and Assay for T cell-mediated cytotoxicity. Primary human T cells were isolated from fresh peripheral blood samples of healthy adult donors (n = 3). Briefly, PBMCs were isolated by density gradient centrifugation using Ficoll Paque PLUS (GE Healthcare Bio-Sciences, Uppsala, Sweden). T cells were isolated using the RosetteSep™ Human CD8+ T Cell Enrichment Cocktail (STEMCELL, Cat#:15023C) following the manufacturer's instructions. Human T cells were maintained in vitro in RPMI-1640 medium supplemented with 5% human serum (ACCESS, Cat.

#:515-HI) and 1% penicillin/streptomycin (Corning, Cat#: 30-002-CI), and 30 units/ml of recombinant human IL-2 (Corning, Cat#:354043). ImmunoCult™ Human CD3/CD28 T Cell Activator (STEMCELL, Cat#:10971) was added to activate the CD8+ T cells. Activated human effector T cells and the indicated tumor cells (3: 1) were cocultured for approximately 72 h. Anti–PD-L1 (durvalumab; MCE, Cat#:HY-P9919), when applicable, were added to the culture media at a final concentration of 10 μg/L. Media were harvested after coculture and subjected to ELISA to detect IL2 (ThermoFisher, Cat#:88-7025-22) and IFN-γ (ThermoFisher, Cat#.88-7316-22) production. Then, tumor cells (GFP+) were determined by Annexin V-APC/7-AAD Apoptosis Detection Kit or stained with crystal violet solution to assess the $CD8^{+}$ T cells-mediated cytotoxicity.

Animal diets.

Mice were fed ad libitum with an irradiated VRFI (P) diet (XIETONG SHENGWU, Cat#1010088) containing about 14.77 kJ/g of gross energy. The FMD diet (ReadyDietech, China) consists of two different components: the day 1 diet and the days 2–4 diet. Day 1 diet contains 7.67 kJ/g (Cat#: RJ20082701) (provided 50% of normal daily intake; 0.46kJ/g protein, 2.2kJ/g carbohydrate, 5.00kJ/g fat); the day 2–4 diet contains 1.48 kJ/g (Cat#: RJ20082702) (provided at 10% of normal daily intake; 0.01kJ/g protein/fat,1.47kJ/g carbohydrates). Mice were transferred in fresh cages to avoid residual chow feeding and coprophagy before the FMD diet was

supplied. Mouse weight loss did not exceed 20% by monitoring daily during the FMD cycle.

Immunohistochemistry

The detailed method was described in our previous study(1,2). The information on antibodies applied in this study as follows: monoclonal antibodies against mouse Ki-67 (Servicebio Cat# GB111499, RRID:AB_2927572) (1:200); Anti-αSMA Antibody (Cell Signaling Technology Cat#19245, RRID:AB) (1:200), EDEM3 (Affinity Biosciences Cat# DF9504, RRID:AB_2842700) (1: 100), PD-L1 (Cell Signaling Technology Cat#13684, RRID:AB _2687655) (1: 200), Anti-F4/80 Antibody (Cell Signaling Technology Cat# 70076, RRID:AB_2799771) (1:200), CD206 (Cell Signaling Technology Cat# 24595, RRID:AB_2892682) (1:200).

Cell viability and Apoptosis assay. Apoptosis was detected using an Annexin V-APC/7-AAD Apoptosis Detection Kit as previously described (MultiSciences, Cat. #:70-AP105) according to the manufacturer's instructions. The results were analyzed using CytExpert software.

PD-L1 and PD-1 binding assay.

The indicated cells were collected, fixed in 4% paraformaldehyde at RT for 15 min, and then incubated with recombinant human PD-1 Fc protein (1μg/100μl per sample)(R&D Systems, Catalog #: 1086-PD) for 1 h. Anti-human IgG H&E/PE (Bioss Cat# bs-0297G-PE) (1: 200) was used as the

secondary antibody. Cells were washed twice with PBS with 2% BSA and resuspended in PBS with 2% BSA. PE fluorescence signal was detected using a CytoFLEX flow cytometer (Beckman Coulter).

Detection of cell surface PD-L1. The indicated cells were collected and then washed with PBS. Next, cells were incubated with PE-conjugated anti-human CD274 antibody (5μl in 100μl PBS per sample) (BD Pharmingen™, Cat#561787) at room temperature for 30 min in the dark. An isotype control was used at the same concentration as the antibody of interest. Then, cells were washed two times with 2mL PBS. Finally, the stained cells were analyzed by CytoFLEX flow cytometer (Beckman Coulter) after resuspension with 400 uL PBS. Data were processed by FlowJo or CytExpert software.

Quantification of cellular UDP-GlcNAc concentrations. The indicated cells (2.5×10^6) were harvested, or cells treated with low glucose for 48h were collected, washed with ice-cold PBS, and lysed with 1mL −80 °C precooled 80% (vol/vol) ethanol. The samples were vortexed for 15 min at room temperature, and then the tube was centrifuged (14,000× g , 10 min, 4 °C), and the supernatant (800μL) was collected. The samples were analyzed by LC/MS. UDP-GlcNAc quantification was made using a calibration curve. Accordingly, the amount of UDP-GlcNAc was divided by the number of cells and the total amount of molecules per 10^6 cells was calculated. The LC/MS was done at the Metabolic Innovation Center of

Sun Yat-Sen University.

Western blot analysis. Cells harvested from the culture were lysed in RIPA-lysis buffer (P0013B, Beyotime, China), and protein concentrations of the samples were determined using the Pierce TM BCA Protein Assay Kit (Thermo Scientific, Waltham, MA, USA). Protein lysates were analyzed by standard SDS/PAGE and transferred to a polyvinylidene fluoride (PVDF) membrane (RIPA) (P0013B, Beyotime, China). Protein bands of interest were revealed by blotting with the respective antibodies. N-linked glycosylated protein was detected by peroxidase-conjugated ConA lectin (1:5000) (L6397, Sigma-Aldrich, USA). Antibody against a-Tubulin (Cell Signaling Technology; Cat#. #2125), antibody against PD-L1 (Cell Signaling Technology Cat#13684, RRID:AB_2687655), antibody against Beta Actin (Proteintech; Cat#. 81115-1-RR), antibody against EDEM3 (Affinity Biosciences Cat# DF9504, RRID:AB_2842700), HK2(Affinity Biosciences Cat# DF6176, RRID:AB_2838143), GFPT1 (Affinity Biosciences Cat# DF8232, RRID:AB_2841529) was used. The density of the protein bands was analyzed using the IMAGEJ software (Bethesda, MD, USA).

Nontarget metabolomics analysis

The indicated cell culture supernatant was collected, and metabolites were extracted[2]. A relevant, fresh medium was used as a control. Briefly, 100μL of the cell culture supernatant was transferred to an EP tube, mixed

with 400 μL of extract solution (50% acetonitrile: 50% methanol); the extraction solution contained deuterated internal standards. The samples were vortexed for 30s, sonicated for 10 min in 4 °C water bath, and incubated for 1 h at -40 °C to precipitate the proteins. Then, the sample was centrifuged at 12000 rpm for 15 min at 4 °C. The resulting supernatant was transferred to a fresh glass vial for analysis. The quality control (QC) sample was prepared by mixing an equal aliquot of the supernatant of samples. This process was performed by Biotree Biomedical Technology Co., Ltd., Shanghai, China.

Animal models and drug treatments. Sex as a biological variable: Our study examined female mice. In some reports, female mice were used to test the effects of FMD (3-5), which may result from female mice exhibit less overall variance and less fighting during fasting.

Female Balb/c nude mice aged 4–6 weeks (~20g) were obtained from Guangdong GemPharmatech Co., Ltd (Guangdong, China). Mice were adapted to a 12 h light/dark cycle and had free access to water. Indicated cells: CT26 $EDEM3^{EV}$ cells ($1*10^6$) and CT26 $EDEM3^{OE}$ cells ($1*10^6$) were implanted subcutaneously into the right flank of immunodeficient nude mice. For the syngeneic model, 6-week-old female BALB/c mice (GemPharmatech Co., Ltd, China) were subcutaneously injected with 8×10^{5} CT26 $EDEM3^{EV}$/$EDEM3^{OE}$ cells resuspended in 100 μL of PBS. Following a 6-day period post-tumor injection, the mice received i.p.

treatments with either 10 mg/Kg anti-PD-L1 antibodies or IgG, along with a FMD supplemented with 1 g/Kg of 2-DG, or a combination of anti-PD-L1 or IgG with FMD and 2-DG. Five or six animals were used in each group. Body weights were recorded daily, and tumor volumes were measured every 3-4 days by a digital vernier caliper according to the following equation: tumor volume (mm^3) =(length × $width^2$) × 0.5. All animals were monitored for abnormal behaviors to minimize animal suffering and pain, and the minimum number of animals necessary for the appropriate sample size was used. Mice were killed when the tumor diameters exceeded 15 mm.

1×10^6 MC38 shCtrl or shEDEM3 cells were injected subcutaneously into 6-week-old C57BL/6J mice in 100 μl of PBS. 7 days after inoculation, mice were randomly divided into different experimental groups (n=8 or 9). Body weights were recorded daily, and tumor volumes were measured every 3-4 days by a digital vernier caliper according to the following equation: tumor volume (mm^3) =(length × $width^2$) × 0.5. All animals were monitored for abnormal behaviors to minimize suffering and pain in the animals, and mice were considered to reach the endpoint when the tumor weight exceeded over 10% of their body weight, or the tumor diameter exceeded 15 mm.

Single-cell RNA-seq analysis

Raw count data were obtained from GSE188711 and GSE236581

processed in Seurat[3] v4 with standard quality control, normalization, scaling, and dimensional reduction steps. Cell annotations from the original study were retained. Major cell types were isolated for separate dimensional reductions.

For pseudo-bulk analysis, we utilized the "aggregateBioVar"[4] package. Raw counts for two epithelial cell subtypes (C91_Epi_Tumor and C90_Epi_MKI67) were extracted and normalized for size factors with "DESeq2"[5] package. These normalized counts were log-transformed, and differences in EDEM3 expression between the different groups were assessed using the Wilcoxon signed-rank test.

Statistical Analysis

All results were expressed as mean ± SD. Student t-tests were performed to compare the differences between the two groups. The difference between growth rates was determined by repeated-measures analysis of variance. All statistical tests were performed using GraphPad Prism 7.0 (GraphPad Software Inc., San Diego, CA), and a 2-tailed P value of 0.05 was considered statistically significant.

Study approval. All patients provided their written informed consent. All animal experiments complied with the ARRIVE guidelines and were operated according to protocols approved by the Institutional Laboratory Animal Care and Use Committee of The Sixth Affiliated Hospital, Sun Yat-sen University, China (IACUC- 2023041701). Ethical approval was gained

from the Institutional Review Board of the Sixth Affiliated Hospital of Sun Yat-sen University, China (2024ZSLYEC-403).

Reference:

1 Peng S, Chen D, Cai J, Yuan Z, Huang B, Li Y *et al*. Enhancing cancer-associated fibroblast fatty acid catabolism within a metabolically challenging tumor microenvironment drives colon cancer peritoneal metastasis. *Mol Oncol* 2021; 15: 1391-1411.

2 Peng S, Li Y, Huang M, Tang G, Xie Y, Chen D *et al*. Metabolomics reveals that CAF-derived lipids promote colorectal cancer peritoneal metastasis by enhancing membrane fluidity. *Int J Biol Sci* 2022; 18: 1912-1932.

3 Hao Y, Hao S, Andersen-Nissen E, Mauck WM, 3rd, Zheng S, Butler A *et al*. Integrated analysis of multimodal single-cell data. *Cell* 2021; 184: 3573-3587.e3529.

4 Thurman AL, Ratcliff JA, Chimenti MS, Pezzulo AA. Differential gene expression analysis for multi-subject single-cell RNA-sequencing studies with aggregateBioVar. *Bioinformatics* 2021; 37: 3243-3251.

5 Love MI, Huber W, Anders S. Moderated estimation of fold change and dispersion for RNA-seq data with DESeq2. *Genome Biol* 2014; 15: 550.

Supplementary Figures and Figure Legends

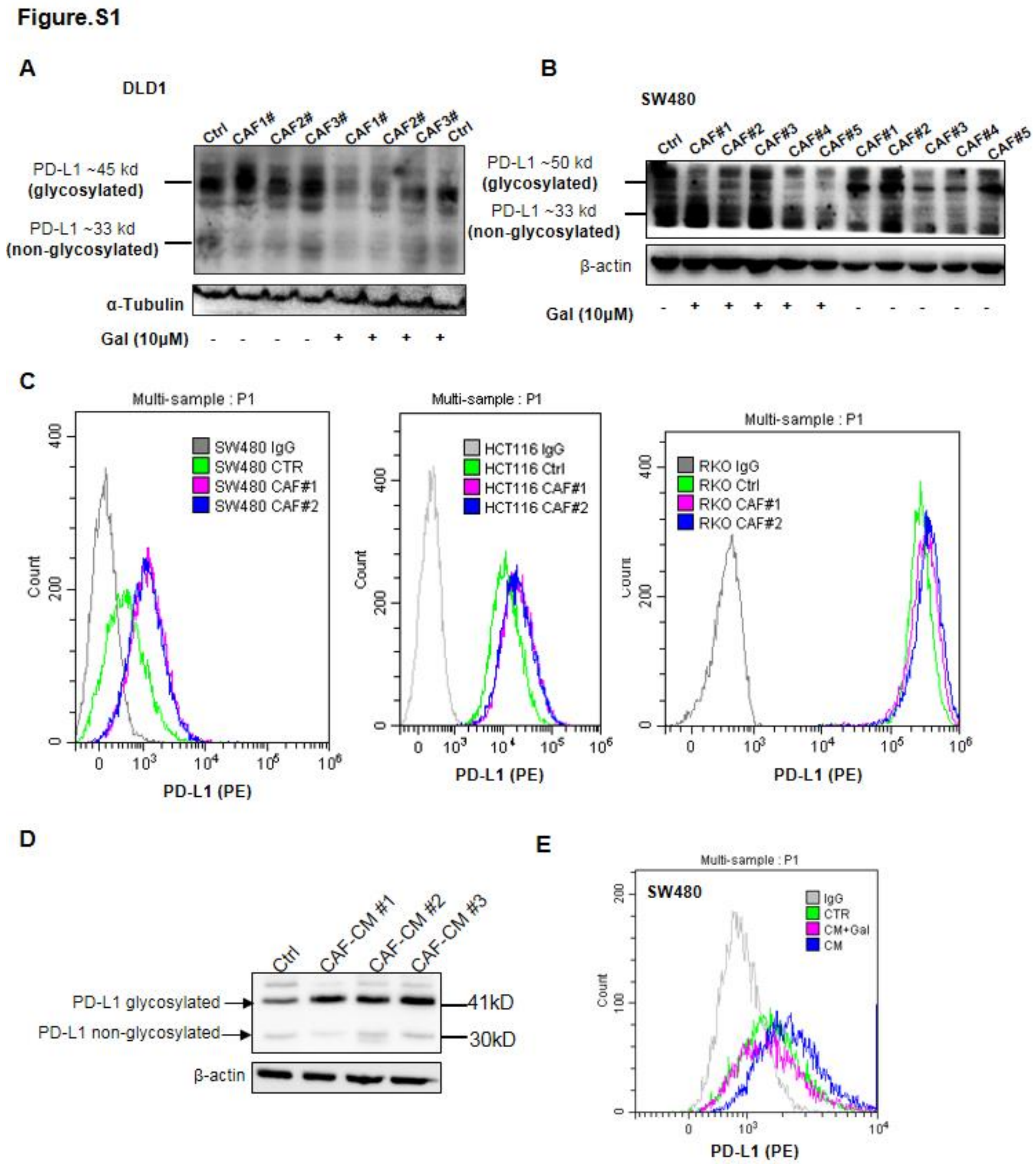

Figure S1. CAFs upregulated the protein expression of PD-L1 glycosylation. (A-B) Immunoblotting analysis of PD-L1 glycosylation and non-glycosylation protein levels in DLD1 (A) and SW480 (B) cells after coculture with CAFs in the presence or absence of 10μM Galunisertib (Gal) for 48h. (C) Flow cytometry analysis of cell surface PD-L1 levels in CRC cell lines after

coculture with CAFs for 48h. (D) Immunoblotting analysis of PD-L1 glycosylation and non-glycosylation protein levels in DLD1 cells upon CAFs-CM treatment for 48h. (E) Flow cytometry analysis of cell surface PD-L1 levels in CRC cell lines upon CAFs-CM or CAFs+Gal-CM treatment for 48h.

Figure. S2

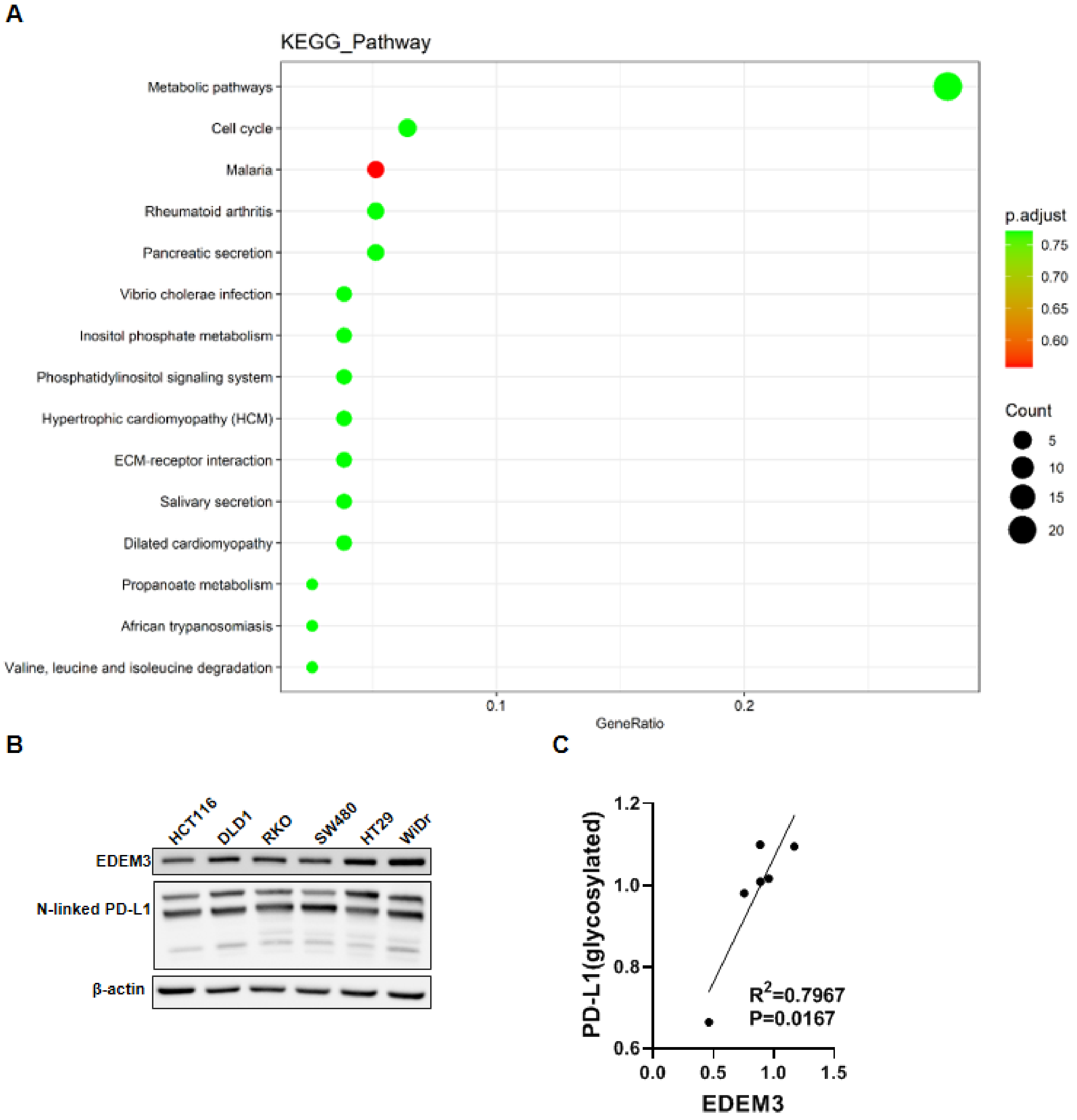

Figure S2. (A) The metabolic pathway was highly enriched in CAF-CM-treated DLD1 cells by proteomics analysis. (B) Immunoblotting analysis of PD-L1 glycosylation and EDEM3 protein levels in 6 CRC cell lines. (C) Correlation analysis of EDEM3 and PD-L1 glycosylation expression. Pearson's correlation test.

Figure. S3

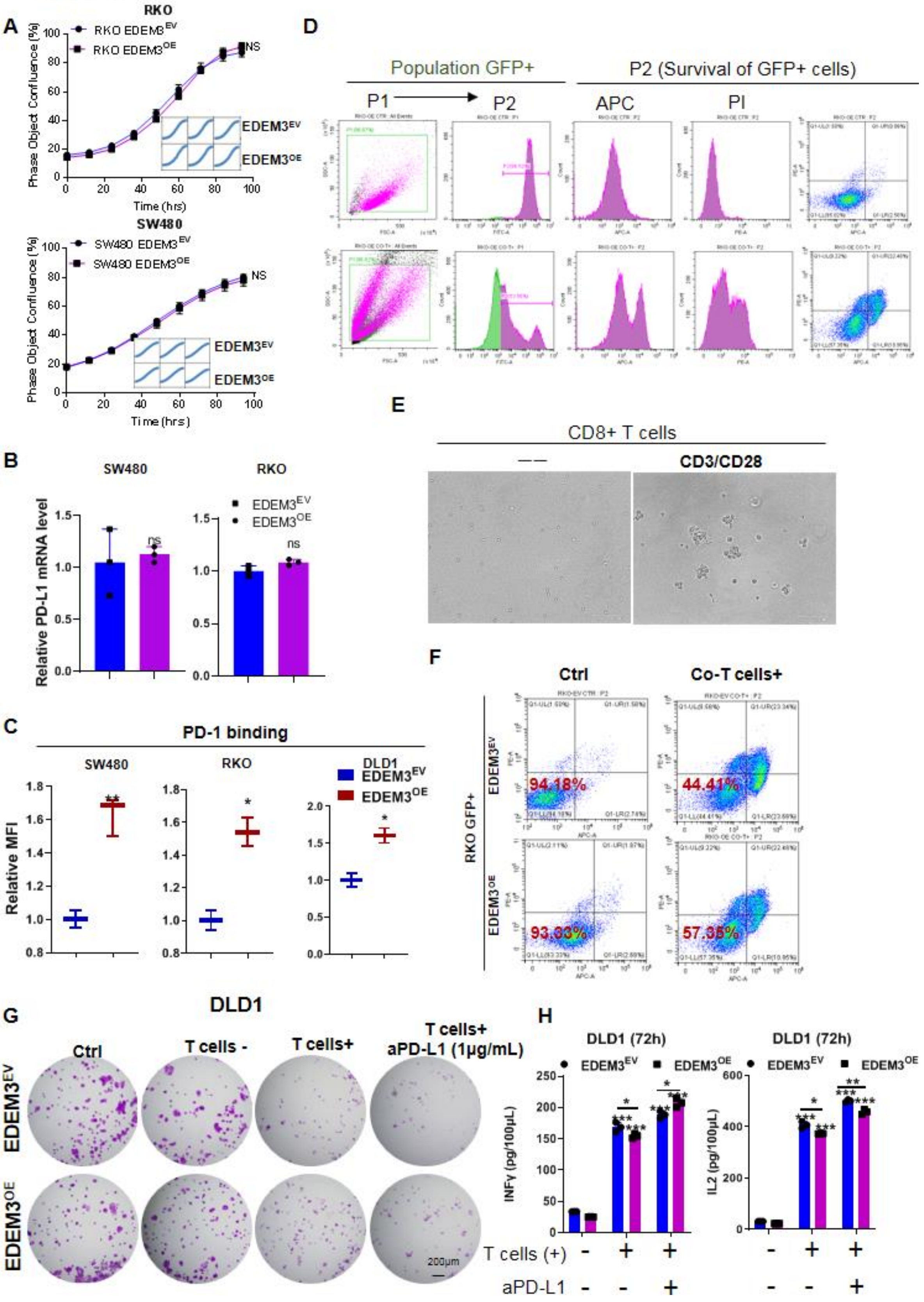
A
RKO
RKO EDEM3EV
RKO EDEM3OE
NS
Phase Object Confluence (%)
Time (hrs)
EDEM3EV
EDEM3OE
SW480
SW480 EDEM3EV
SW480 EDEM3OE
B
SW480
RKO
EDEM3EV
EDEM3OE
Relative PD-L1 mRNA level
ns
C
PD-1 binding
SW480
RKO
DLD1
Relative MFI
D
Population GFP+
P2 (Survival of GFP+ cells)
P1
P2
APC
PI
E
CD8+ T cells
CD3/CD28
F
Ctrl
Co-T cells+
RKO GFP+
94.18%
44.41%
93.33%
57.35%
G
DLD1
Ctrl
T cells -
T cells+
T cells+ aPD-L1 (1μg/mL)
200μm
H
DLD1 (72h)
INFγ (pg/100μL)
IL2 (pg/100μL)
T cells (+)
aPD-L1

Figure S3. EDEM3 upregulates PD-L1 glycosylation, increases cell-surface PD-L1 and limits T cell-dependent antitumor immunity. (A) Growth curves for the indicated cells were measured by the IncuCyte ZOOM® live−cell imager. n = 3. Two-way ANOVA. (B) Relative PD-L1 mRNA expression levels were measured with RT-qPCR. n=3, Unpaired Students t-test. (C) FACS gates for the GFP+ and AnnexinV-/PI- subsets are shown. (D) The activated CD8 + T cells were imaged by microscopy. (E) The binding of PD-1 to PD-L1 in indicated cells was quantified as in Figure 3F. Unpaired Students t-test. (F) Cell apoptosis (GFP+) upon co-culture with activated T cells was determined by flow cytometry. $EDEM3^{EV}$ and $EDEM3^{OE}$ cells were cotransfected with GFP; consequently, apoptosis was assessed specifically in the GFP+ cell populations. Percentages of cell survival are shown. (G) The survival cells after treatment under the indicated conditions were shown by crystal violet staining. Representative of two experiments was shown. (H) Interferon (IFN)-gamma and interleukin (IL)-2 cytokine expression was measured by ELISA. n = 2 independent assays, obtained from different T cells isolated from 2 health donors, each arising from three quantification replicates. Unpaired Students t-test. All data are shown as mean ± SD; *P < 0.05, **P < 0.01, ***P < 0.001.

Figure.S4

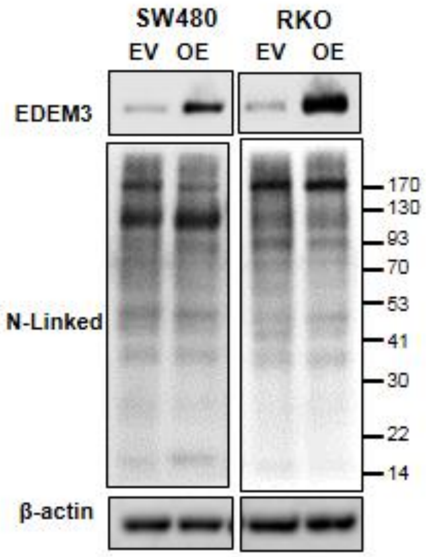

Figure S4. Immunoblotting analysis of global N-glycosylation levels in EDEM3^{OE} and EDEM3^{VC} cells.

Figure. S5

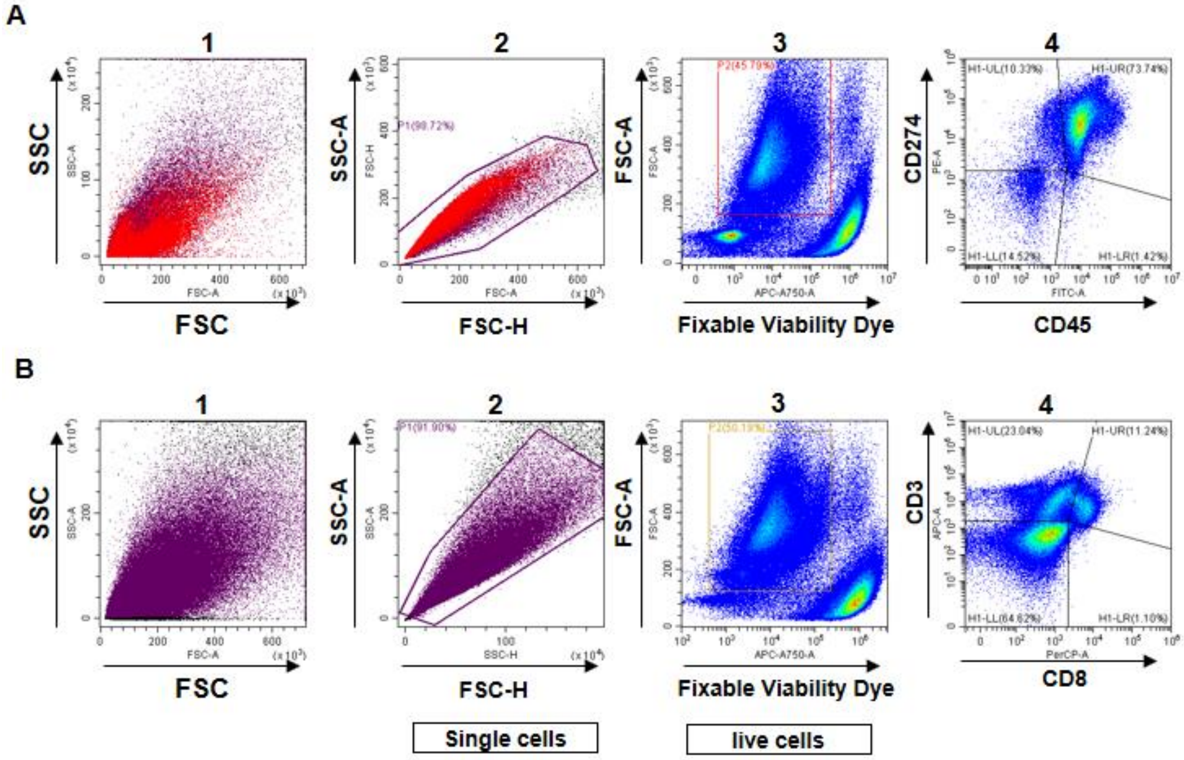

Figure S5. Gating strategy for sorting $CD45^-/CD274^+$ cells (A) and $CD3^+/CD8^+$ cells (B) by FACS.

Figure. S6

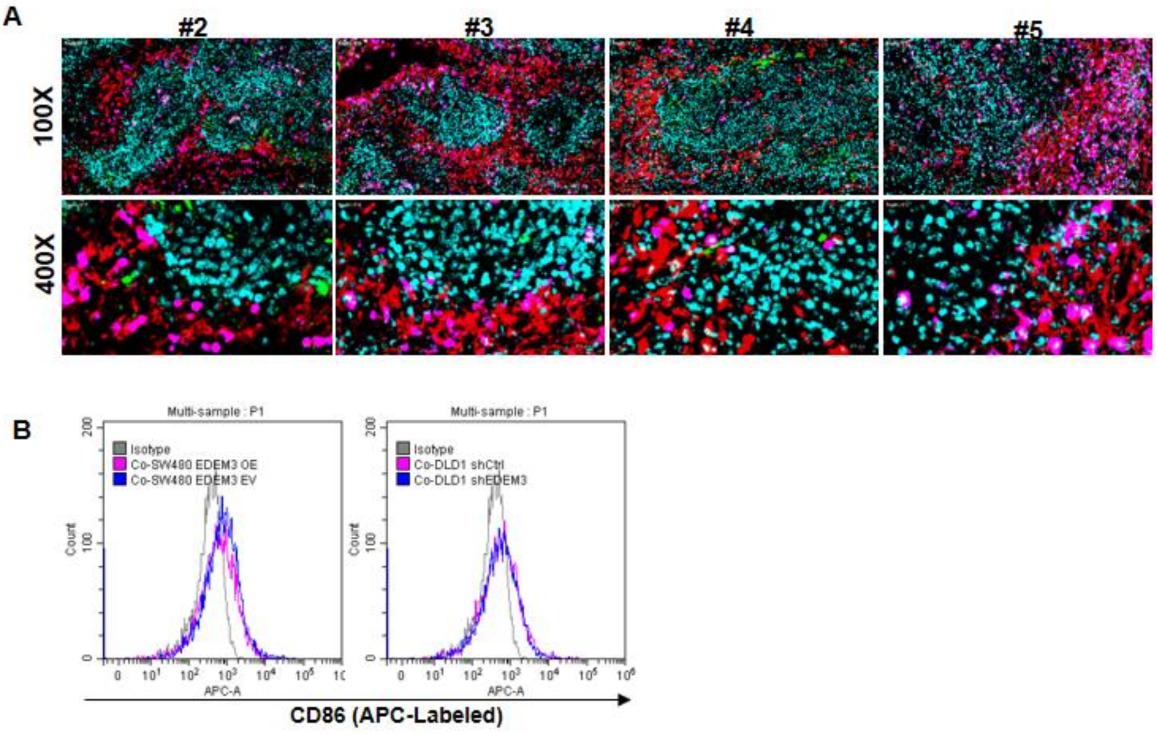

Figure S6. EDEM3 accelerates PD-1 antibody treatment resistance by recruiting M2-like macrophages. (A) Individual $EDEM3^{OE}$ tumors from different mice after anti-PD-1 treatment were immunostained by Multiplex IHC with anti-CD8, CD206 and α-SMA as indicated. (B) FACS analysis determined $CD86^+$ M1 macrophages.

Figure. S7

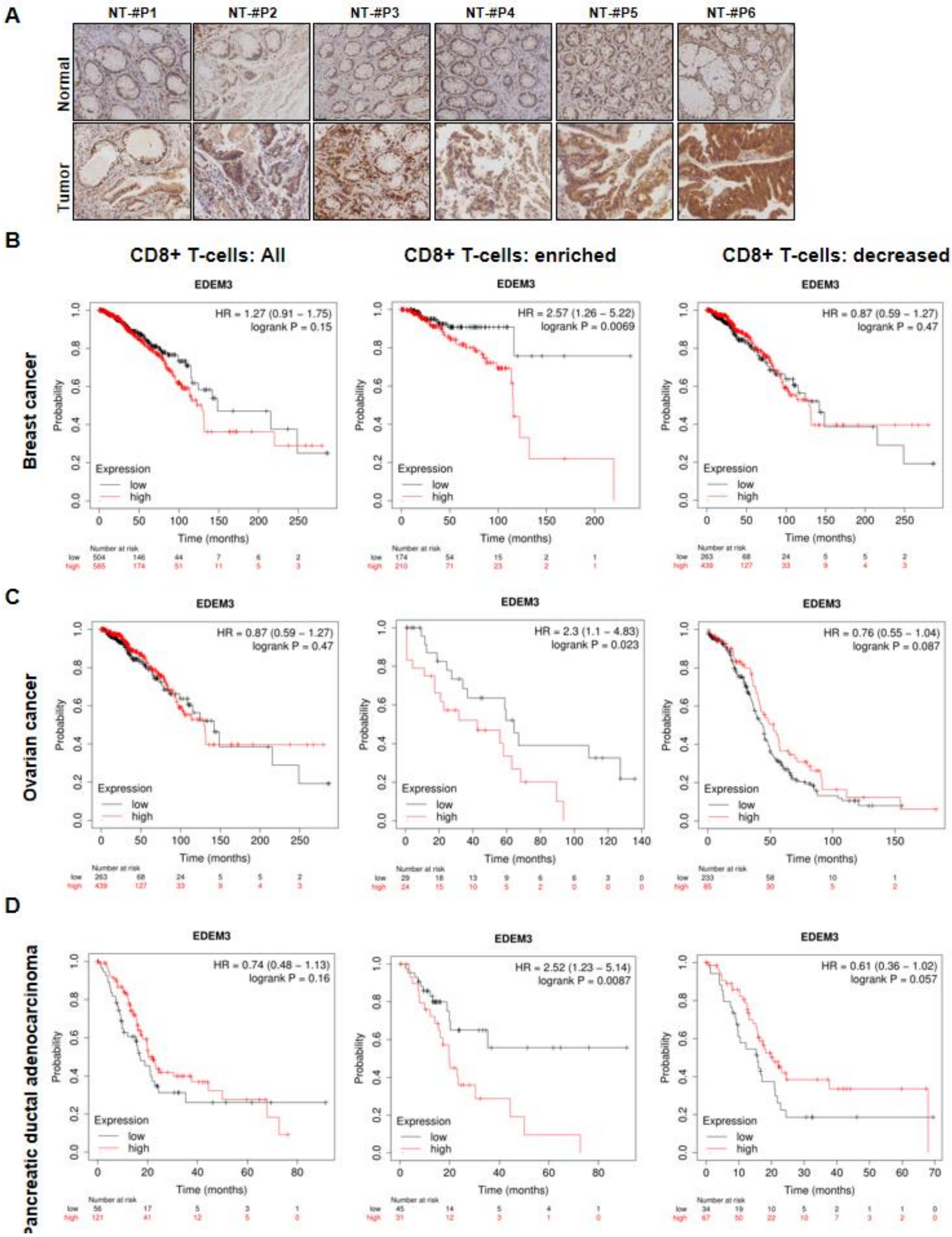

Figure S7. EDEM3 expression correlates with prognosis and immunogenicity in CRC cancer. (A) Immunohistochemistry analysis of EDEM3 expression was performed on the same colorectal tumor and

normal tissue samples. (B-D) Kaplan–Meier overall survival curves of Breast cancer (B), Ovarian cancer (C), and Pancreatic ductal adenocarcinoma (D) patients with high and low EDEM3 mRNA expression with all (left panel), enriched (middle panel) and decreased $CD8^+$ T cells (right panel) obtained from the online Kaplan-Meier Plotter database (http://kmplot.com/analysis). Statistical analyses were performed by the two-tailed log-rank test.

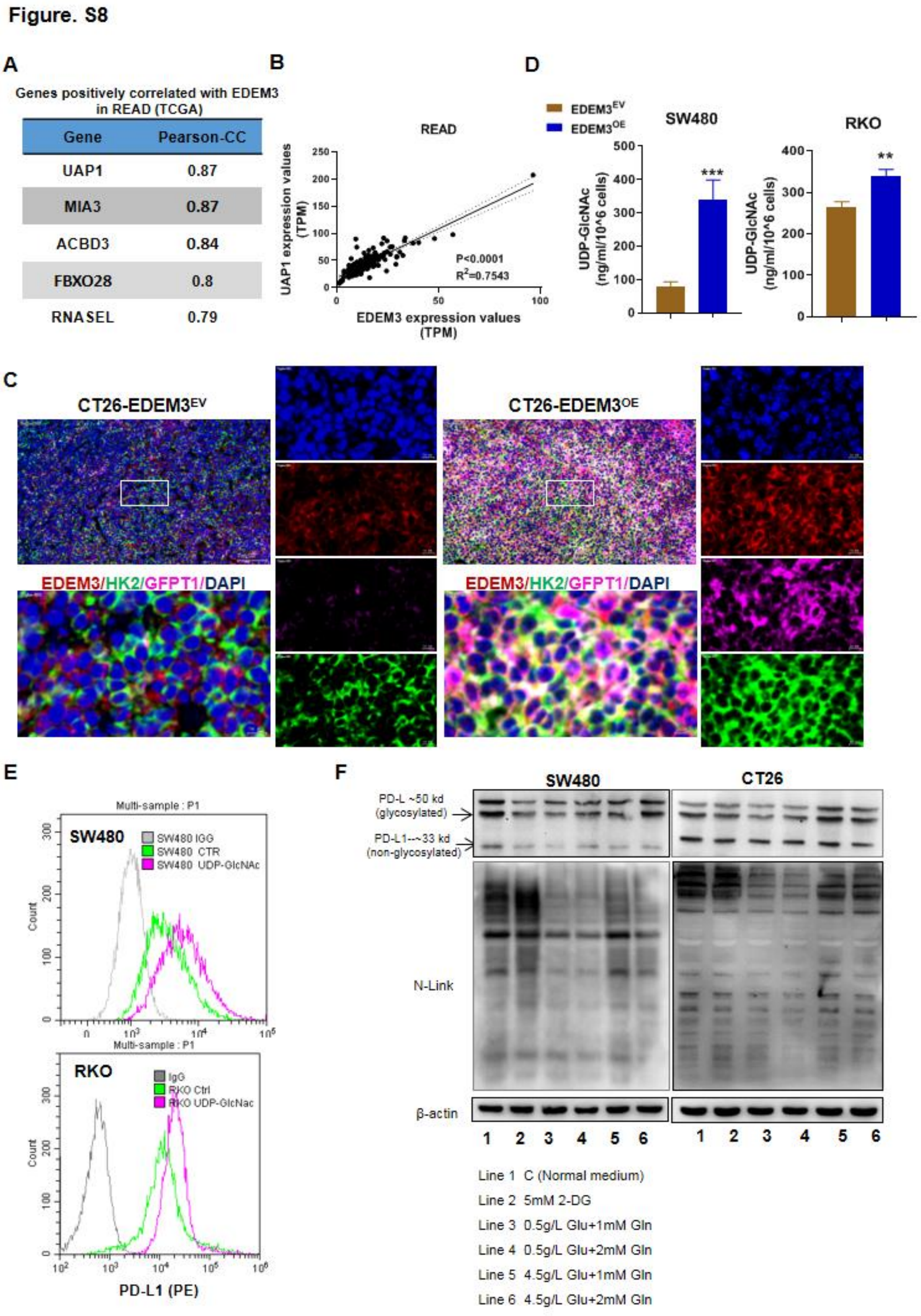

Figure S8. Glucose deprivation decreases global N-Glcosylation and cell-surface PD-L1 levels in CRC cells. (A-B) Correlations were

performed in READ by Spearman correlation test based on the TCGA databases. Pearson's correlation test. (C). Representative mIHC image of EDEM3, HK2, GFPT1 with DAPI staining in $EDEM3^{EV}$ and $EDEM3^{OE}$ tumors. Raw mIHC data are shown in Supplementary Figure S9. (D) UDP-GlcNAc concentrations measured by LC-MS/MS (*n*=3). Unpaired Students t-test. (E) Flow cytometric analysis of PD-L1 expression on cell surface after the indicated treatment. (F) Immunoblotting analysis of global N-glycosylation and PD-L1 glycosylation levels in DLD1 and CT26 cells treated with medium containing different levels of glucose and glutamine or 2-DG (5mM) for 48 hours. Line 1: C (Normal medium), Line 2: C+5mM 2-DG, Line 3: 0.5g/L Glu+1mM Gln, Line 4: 0.5g/L Glu+2mM Gln, Line 5: 4.5g/L Glu+1mM Gln, Line 6: 4.5g/L Glu+2mM Gln. All data are shown as mean ± SD; ***P < 0.001.

Figure. S9

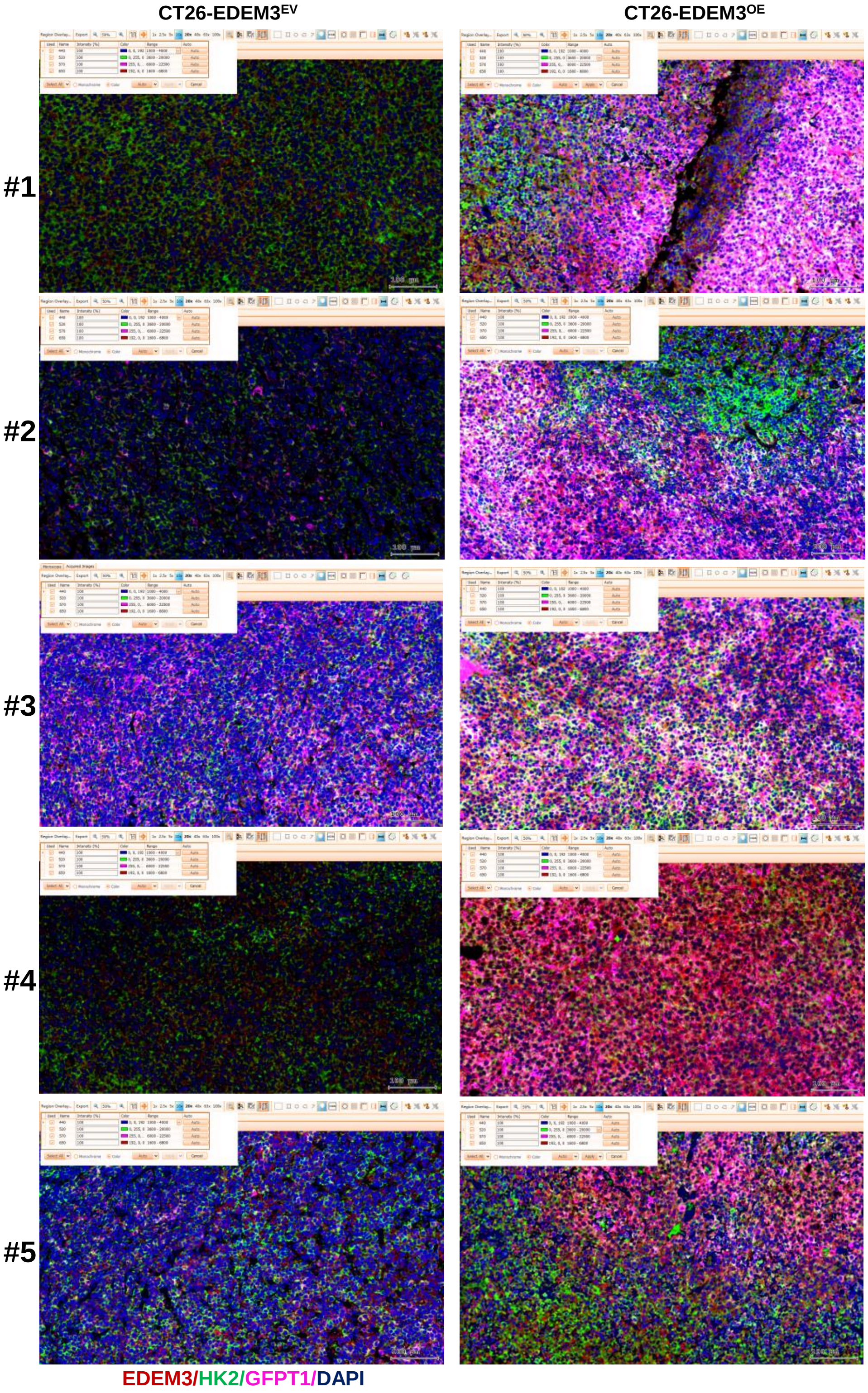

Figure S9. Representative mIHC image of EDEM3, HK2, GFPT1 with DAPI staining in EDEM3^{EV} and EDEM3^{OE} tumors. Each image represents

one tumor coming from one independent mouse. Shown are the same fluorescence intensity values.

Figure. S10

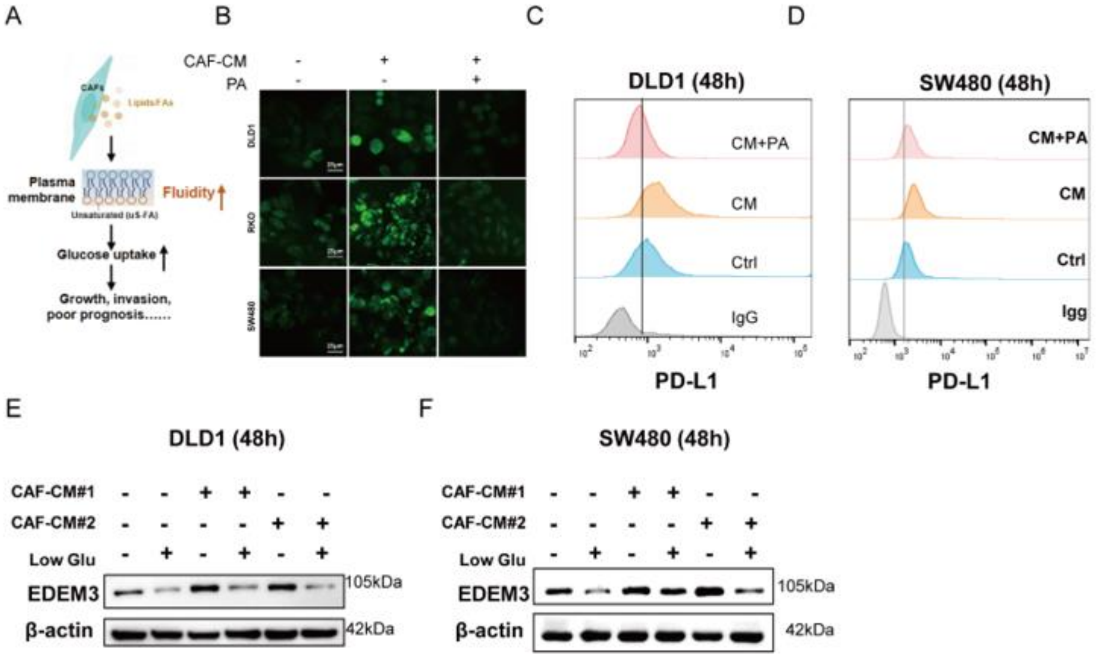

Figure S10. CAFs increased EDEM3 expression in CRC cells as a result of enhanced exogenous glucose uptake. (A) Schematic of the mechanism of CAFs enhances glucose uptake by increasing cell membrane fluidity. (B) Glucose uptake in DLD1 and RKO cells incubated with indicated treatment visualized by 2-NBDG fluorescence. n=2. PA: 50μM (C-D) Flow cytometric analysis of PD-L1 expression on DLD1 (C) and SW480 cells (D) surface after the indicated treatment. PA: 50μM. (E-F) Immunoblotting analysis of PD-L1 glycosylation and EDEM3 levels in

DLD1 (E) and SW480 cells (F) treated with normal glucose (4.5 g/L) or low glucose (1g/L) under the indicated conditions for 48 hours.

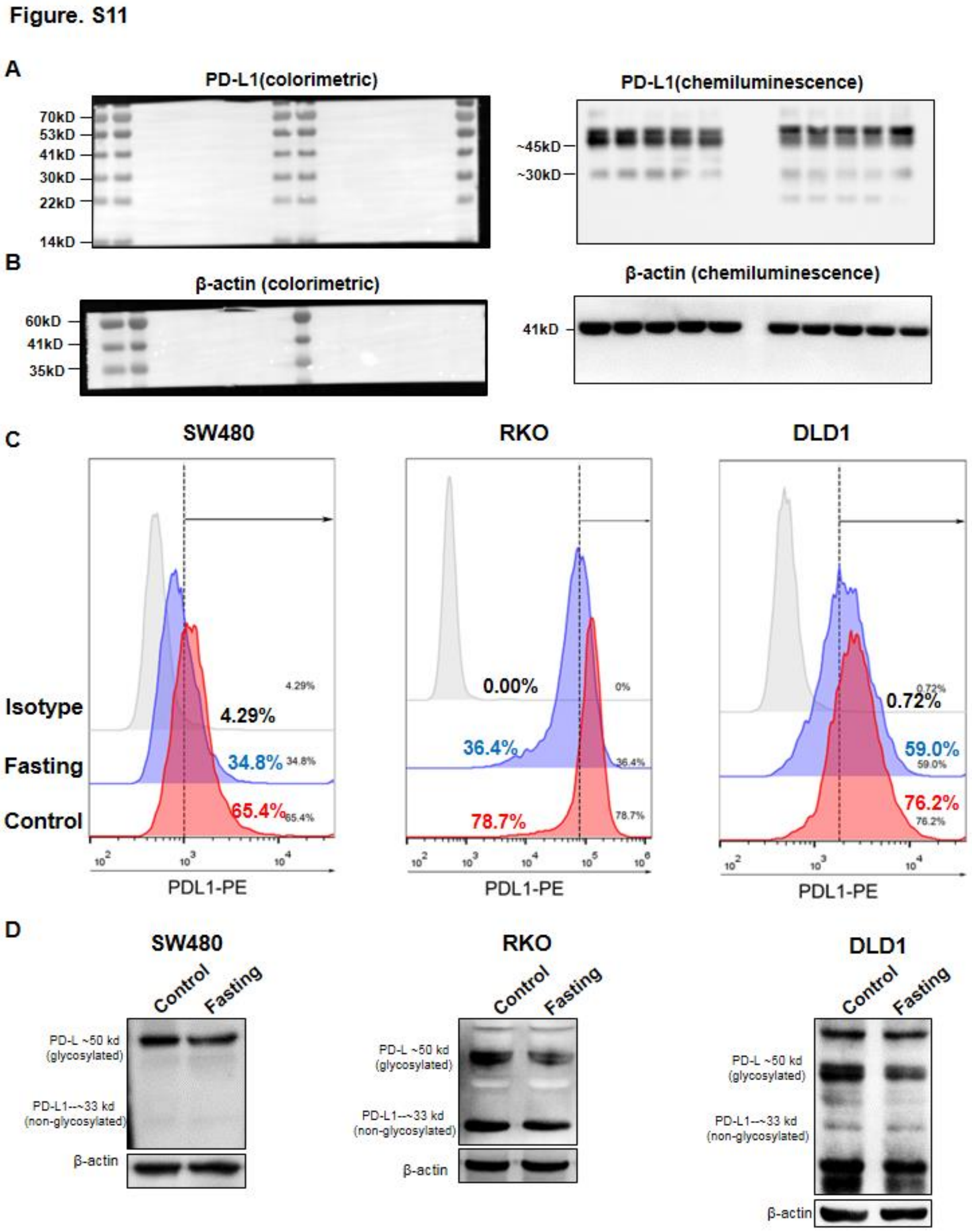

Figure S11. Images of Western blot raw data. (A) Uncropped Western Blot data of PD-L1. (B) Uncropped Western Blot data of β-actin. (C) Flow

cytometry analysis of cell surface PD-L1 levels in indicated cells after fasting treatment for 48 hours. (D) Immunoblotting analysis of PD-L1 glycosylation and non-glycosylation protein levels in indicated cells after fasting treatment for 48 hours.

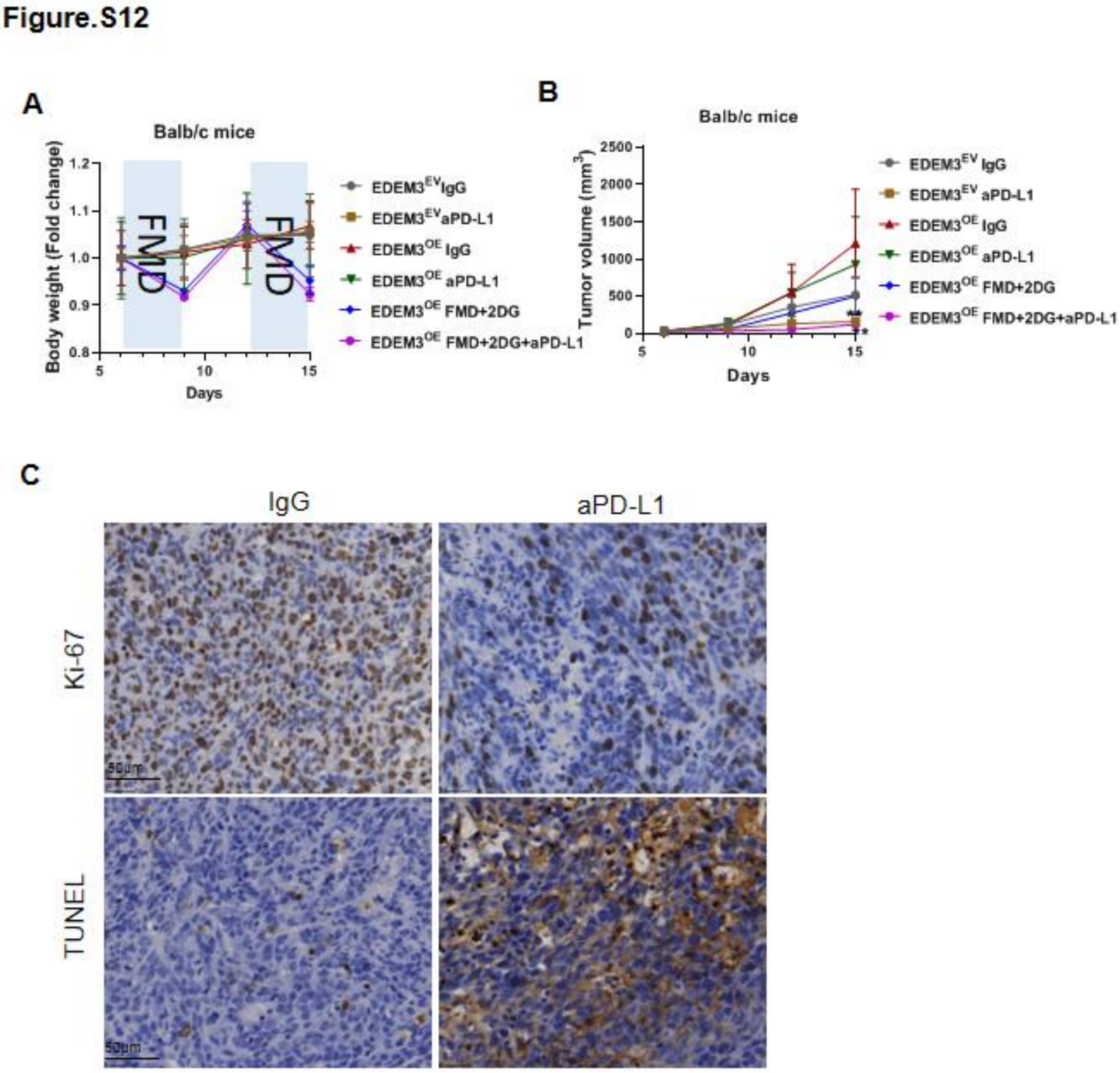

Figure S12. Glucose restriction produces an enhanced antitumor effect and ablates PD-1/PD-L1 blockade resistance in EDEM3-high expression tumors. (A-B) Balb/c mice bearing CT26 xenograft tumors

were fed ad libitum or subjected to FMD cycles and treated with the indicated agents. n = 5 per group. Body weight(A) and Tumor growth (B) are presented. (C) Immunohistochemical results of Ki67 and TUNEL expression in the respective group were shown. All data are shown as mean ± SD; *P < 0.05, **P < 0.01.
